# Aminal‐linked Covalent Organic Frameworks for Light Energy Upconversion

**DOI:** 10.1002/anie.202522521

**Published:** 2026-02-23

**Authors:** Mateusz Brzeziński, Agata Tyszka‐Gumkowska, Aleksander Gorski, Marek J. Potrzebowski, Tomasz Polczyk, Wojciech Wegner, Sylwester Gawinkowski, Jakub Ostapko

**Affiliations:** ^1^ Institute of Physical Chemistry Polish Academy of Sciences Warsaw Poland; ^2^ Centre of Excellence ENSEMBLE3 Sp. z o.o. Warsaw Poland; ^3^ Centre of Molecular and Macromolecular Studies Polish Academy of Sciences Łódź Poland; ^4^ Faculty of Chemistry Jagiellonian University Kraków Poland

**Keywords:** aminal‐linked COFs, covalent organic frameworks, photon upconversion, triplet migration, triplet‐triplet annihilation

## Abstract

The conversion of two low‐energy photons into a single higher‐energy photon at low irradiance is highly desirable for bioimaging and solar energy harvesting. Yet translating established solution‐phase triplet–triplet annihilation upconversion (TTA‐UC) systems into robust solid‐state platforms remains a challenge. Here, we report the first covalent organic frameworks (COFs) capable of sensitized TTA‐UC. Two aminal‐linked frameworks integrating anthracene chromophores, **Ant‐COF‐H** and **Ant‐COF‐OH**, were synthesized and structurally characterized, revealing high crystallinity and strong photoluminescence (*Φ_F_* ≈ 40%). When sensitized with a palladium porphyrin complex, both COFs display upconverted emission with quantum yields up to 1.8%, surpassing the performance of the conventional all‐in‐solution reference system. Notably, the onset of saturation occurs at excitation power densities as low as 100 mW cm^−2^. Time‐resolved emission spectroscopy reveals fast energy‐transfer consistent with intra‐framework triplet migration rather than diffusion. Finally, we correlate framework structural features with energy‐loss pathways, providing design guidelines for further improvement. This work establishes a foundation for practical, low‐power light management in crystalline polymers by demonstrating that aminal‐linked COFs can be engineered to support efficient energy transfer and function as solid‐state upconverters.

## Graphical abstract

Aminal‐linked covalent organic frameworks incorporating anthracene units are introduced as solid‐state platforms for triplet–triplet annihilation upconversion. The crystalline materials retain chromophore electronic properties and exhibit fluorescence quantum yields of up to 40%. Internal energy transfer processes, governed by the alignment of anthracene units within the framework, enable upconversion with quantum yields of 1.8% under low‐intensity excitation. This work demostrates, for the first time, TTA upconversion in COFs, opening new avenues for energy conversion and optoelectronic applications.

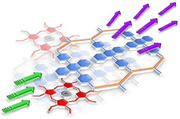

## 1 Introduction

Upconversion, in which two low‐energy photons combine to yield one higher‐energy photon, has wide‐ranging practical uses. In bioimaging, near‐infrared (NIR) excitation upconverted to visible emission reduces phototoxicity and decreases autofluorescence [1, 2]. In photovoltaics, converting sub‐bandgap NIR sunlight into usable photons recovers otherwise wasted light and boosts efficiency [3, 4, 5].

Photon upconversion can be achieved through several approaches, including two‐photon absorption [6], energy transfer upconversion with lanthanide‐based systems [7, 8], and sensitized triplet–triplet annihilation (sTTA) [9, 10]. Among these, sTTA uniquely combines excitation and emission wavelength tunability with efficient operation under low excitation power densities comparable to solar radiation, making it particularly suitable for biological and light‐harvesting applications [11, 12, 13].

**FIGURE 1:**
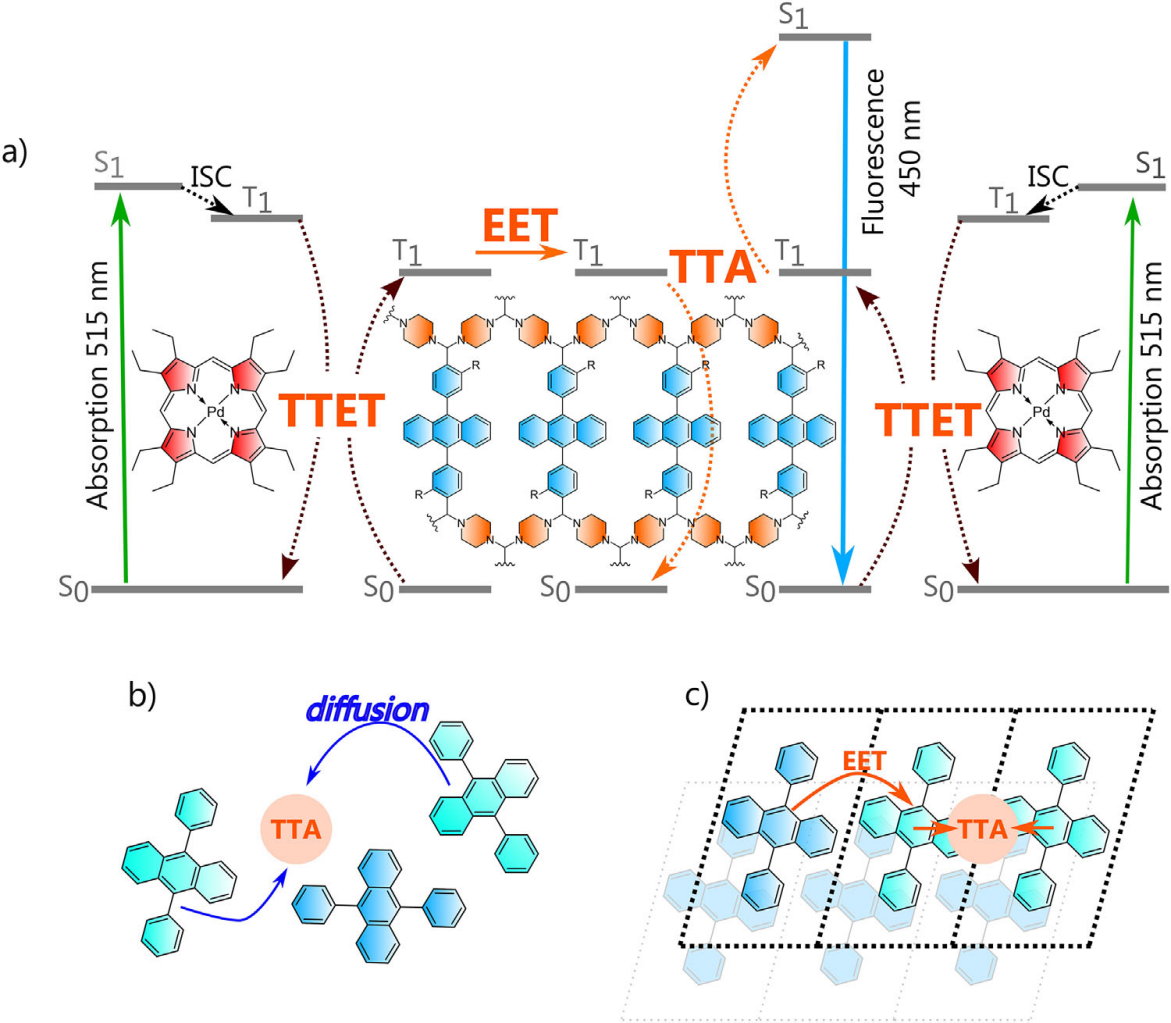
a) Simplified Jablonski diagram for the sTTA upconversion process with triplet‐triplet energy transfer (TTET) occurring between dissolved porphyrin and polymer suspension, exciton energy transfer (EET) within the polymer framework, and triplet‐triplet annihilation (TTA) occurring between emitter units incorporated into the polymer framework, b) upconversion in solution driven by the diffusion of excited emitter molecules, c) upconversion in solid state driven by the EET between emitter units in the polymeric network.

The sTTA upconversion process requires two components to interact: a photosensitizer and an emitter (Figure 1a). The photosensitizer absorbs low‐energy light and, after intersystem crossing, populates its first excited triplet state (T_1_). An emitter, also referred to as an annihilator, is excited to its triplet state through energy transfer from the photosensitizer (triplet–triplet energy transfer, TTET). When two emitter triplet excitons meet, they undergo bimolecular triplet–triplet annihilation (TTA), typically resulting in one molecule returning to the ground state (S_0_) and the other transitioning to an electronically excited singlet state (S_1_). This singlet state relaxes radiatively, releasing upconverted emission [10]. Overall, the process requires two photons for excitation, but one photon is released as upconverted radiation, which limits the external quantum yield to 50%.

The most straightforward upconverting systems consist of co‐dissolved photoactive components, where performance of photon upconversion is governed by molecular diffusion (Figure 1b). However, the inherent instability of solution‐based systems makes them unsuitable for scalable processing and commercialization, motivating the development of more robust solid‐state upconverters. Transitioning from solution to solid state presents yet another major challenge [14, 15]. In the solid state, translational diffusion is suppressed and upconversion relies on excited‐state energy migration—exciton energy transfer (EET)—between neighboring molecular units (Figure 1c) [16, 17]. This change in mechanism demands a delicate balance: active units must be positioned closely enough and suitably oriented to enable efficient triplet–exciton hopping yet sufficiently separated to prevent aggregation‐induced quenching and non‐radiative trapping [17].

To address these structural requirements, porous metal‐organic frameworks (MOFs) have emerged as promising platforms for solid‐state upconversion. These materials can be engineered with photoactive units at well‐defined orientations and separations, making them ideal candidates for maintaining efficient excited state energy migration. Their intrinsic porosity separates the photoactive components, while the nodes used in framework construction predefine spatial arrangements [18, 19, 20, 21, 22, 23]. However, the presence of metallic centers in MOFs raises concerns about limited stability and potential toxicity, which negatively affect their prospects for further implementation in biological and light‐harvesting systems.

Covalent organic frameworks (COFs) combine the advantages of MOFs with superior structural stability and non‐toxicity. These crystalline, reticular systems, first introduced in 2005 by Yaghi [24], are composed entirely of organic units assembled *via* the reversible formation of covalent bonds. The non‐metallic nature of COFs mitigates issues related to toxicity and material instability [25]. High crystallinity of COFs further enables precise control over the geometrical alignment of built‐in photoactive units, in contrast to the related amorphous polymers [26, 27, 28]. Additionally, the absence of metallic centers, which often facilitate rapid non‐radiative recombination [29, 30, 31, 32], is an advantage that distinguishes COFs from MOF‐based systems for emissive applications.

In recent years, significant progress has been made in developing highly emissive COFs [33, 34, 35, 36]. Among the various types reported, aminal‐linked frameworks are particularly attractive owing to their cost‐effective synthesis from inexpensive piperazine and readily available aldehydes. In contrast to the well‐established imine‐linked COFs, their formation proceeds without the need for a catalyst [37, 38]. Furthermore, the aliphatic character of piperazine suppresses in‐plane π–π conjugation and weakens interlayer interactions, thereby enabling embedded chromophores to retain their intrinsic molecular properties [33, 36, 39]. Encouraged by these features, we explored the potential of aminal‐linked COFs as functional materials for photon upconversion.

Here, we report the synthesis and characterization of two aminal‐linked COFs, **Ant‐COF‐H** and **Ant‐COF‐OH**. Both materials incorporate 9,10‐diphenylanthracene (DPA) as emissive units, while the aliphatic piperazine‐based linkage suppresses interlayer interactions and preserves the intrinsic emissive properties of the DPA chromophore, yielding strong photoluminescence with quantum yield of about 40%. Remarkably, both COFs undergo sensitized triplet–triplet annihilation upconversion (sTTA‐UC) using palladium(II) 2,3,7,8,12,13,17,18‐octaethylporphyrin (PdOEP) as the sensitizer. In the proposed upconversion scheme, the sensitizer is not an integral component of the framework but an external species that is functionally decoupled from the COF scaffold, enabling the isolation of framework‐related processes and, in principle, its replacement by metal‐free alternatives. The achieved upconversion quantum yield (*Φ_UC_*) of up to 1.8% surpasses most values reported for MOF‐based systems and represents, to the best of our knowledge, the first successful application of COF materials in photon upconversion.

## 2 Results and Discussion

### 2.1 Synthesis

Diphenylanthracene (DPA) is a prototypical emitter for photon upconversion and is most commonly studied in solution, where its photophysical properties are well established [40, 41], including its demonstrated role as a triplet energy acceptor when excited by the PdOEP as the photosensitizer [40, 42]. To introduce the DPA unit into the polymer framework, the DPA carbaldehyde derivatives **Ant‐CHO** and **Ant‐CHO(OH)** were synthesized by classical Suzuki reaction approach (Section S2).

**FIGURE 2:**
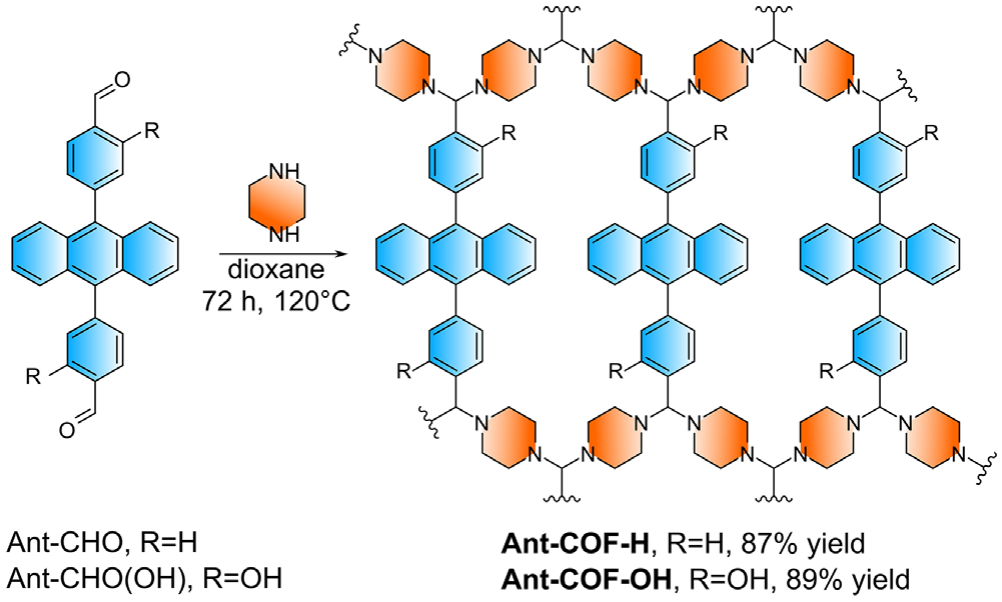
Solvothermal synthesis of **Ant‐COF‐H** and **Ant‐COF‐OH** by condensation of **Ant‐CHO** and **Ant‐CHO(OH)** with piperazine.

Notably, **Ant‐CHO(OH)** features hydroxyl groups introduced for two purposes: to modulate the energetics of singlet and triplet states, and to assess their impact on material crystallinity, potentially forming intramolecular hydrogen bonds with aminal nitrogen atoms. The synthesized building blocks, **Ant‐CHO** and **Ant‐CHO(OH)**, were subjected to reaction with piperazine under optimized conditions to form reticular polymers with high yields (Figure 2). Details of optimization process are provided in the Supporting Information (Section S3). The products were isolated with defined yields and characterized for crystallinity (PXRD) as key metrics (Section S3).

### 2.2 Material Characteristics

**FIGURE 3:**
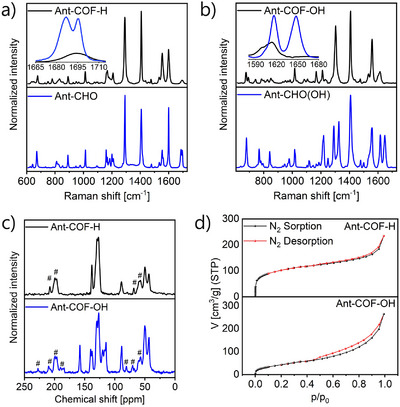
Raman spectra of synthesized COFs and used aldehyde substrates a) for **Ant‐COF‐H**, b) for **Ant‐COF‐OH**; c) ^13^C solid state NMR spectra of **Ant‐COF‐H** and **Ant‐COF‐OH**, # denotes spinning sidebands; d) nitrogen sorption and desorption isotherms used to calculate the BET surface area.

The formation of **Ant‐COF‐H** and **Ant‐COF‐OH** has been confirmed by Raman, infrared, and solid‐state ^13^C CP/MAS NMR spectroscopies. The Raman spectra of aldehyde substrates reveal closely spaced bands with maxima at 1687 cm^−1^ and 1695 cm^−1^ for **Ant‐CHO**, and at 1682 cm^−1^ and 1693 cm^−1^ for **Ant‐CHO(OH)** (Figure 3a and b). In each system investigated, one of the bands disappears upon formation of the aminal bonds, which is associated with the transformation of the carbonyl group into an aminal linkage. These results are consistent with the IR data (Figure S7). For both **Ant‐COF‐H** and **Ant‐COF‐OH** the ^13^C CP/MAS NMR spectra (Figure 3c) reveal peaks in the 145–115 ppm range assigned to the sp^2^ carbons of phenyl and anthracene rings. In both spectra signals of sp^3^ carbons assigned to the piperazine rings and located at 40–55 ppm, and sp^3^ carbon signal assigned to atoms linked directly with nitrogen and located at 89 ppm are present. A characteristic signal at 158 ppm in the **Ant‐COF‐OH** spectra assigned to the sp^2^ carbon bonded with the hydroxyl group was also observed.

Powder X‐ray diffraction (PXRD) patterns obtained for **Ant‐COF‐H** and **Ant‐COF‐OH** confirm the crystalline nature of these materials (Figures 5a and 6a). Both COFs display comparable sets of diffraction peaks, although those of **Ant‐COF‐OH** are noticeably narrower. This suggests that introducing the hydroxyl group improves material crystallinity but does not cause significant changes to the lattice parameters or atomic positions.

**FIGURE 4:**
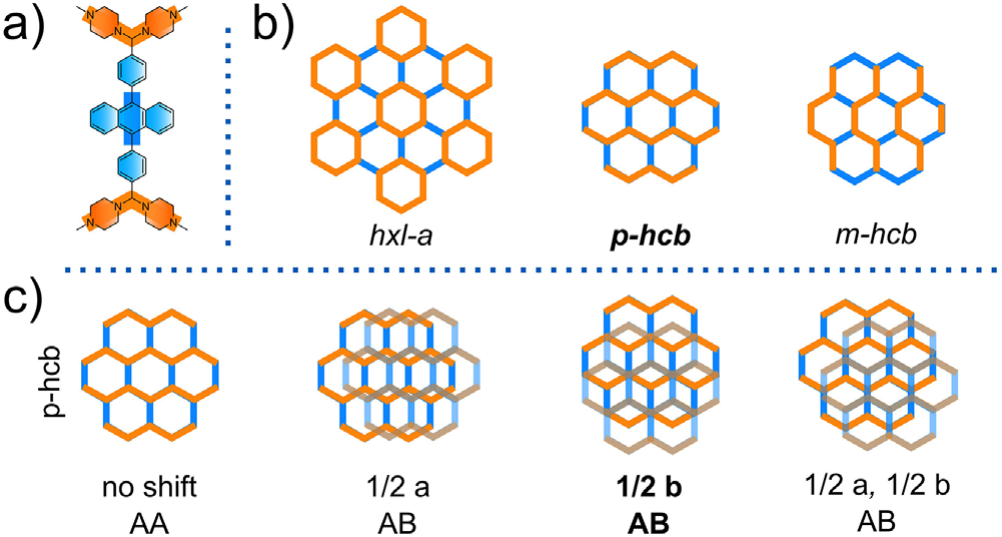
a) Structure‐symbol assignment, b) considered topologies of investigated COFs, c) considered stacking modes for the *p‐hcb* topology.

To elucidate the structures of **Ant‐COF‐H** and **Ant‐COF‐OH**, periodic models were constructed for both systems, considering three possible topologies (*hxl‐a*, *p‐hcb*, *m‐hcb*) and different stacking modes (AA and three AB stacking variants for each topology). The nomenclature of topologies was adopted from Hong et al. [43]. The studied topologies are schematically illustrated in Figure 4. All models were evaluated by comparing simulated PXRD patterns with experimental data (Sections S1 and S5).

**FIGURE 5:**
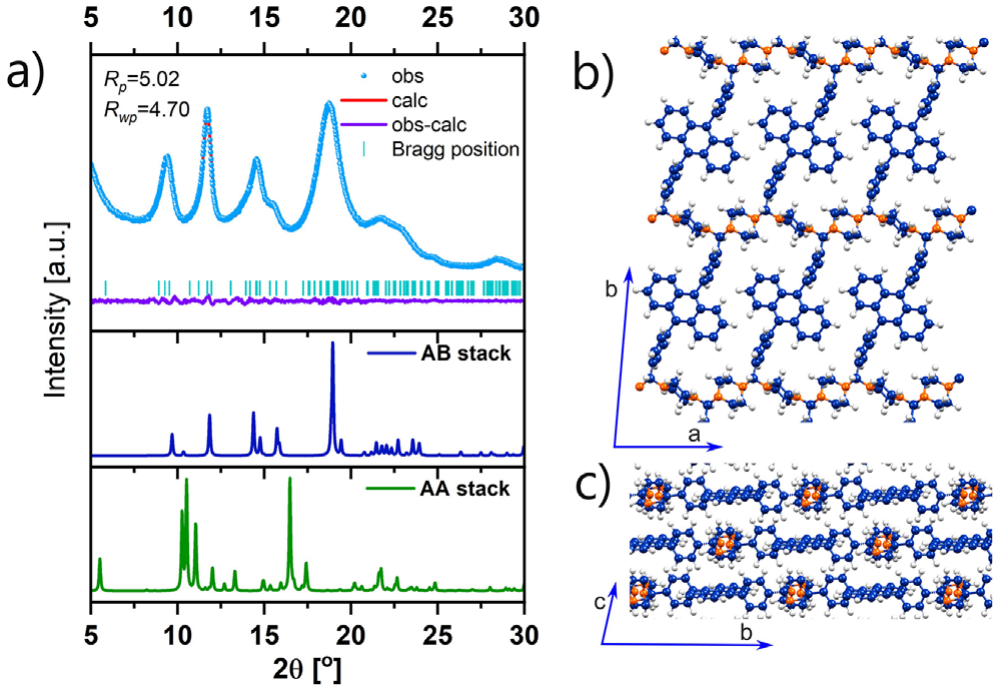
a) Experimental PXRD pattern of **Ant‐COF‐H** (blue dots), Le‐Bail refined profile (red line), difference between experimental and refined pattern (violet line), refined positions of Bragg reflections (blue line symbols), calculated PXRD patterns for simulated AB‐type stack (deep blue line) and AA‐type stack (green line) of **Ant‐COF‐H**. Calculated structure of **Ant‐COF‐H**: b) view along the c‐axis, single layer shown, c) view along the a‐axis.

**FIGURE 6:**
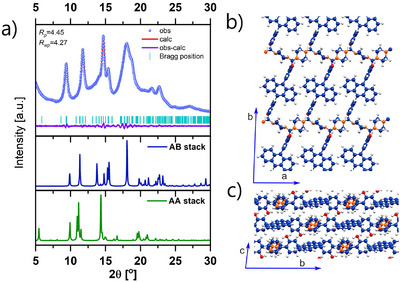
a) Experimental PXRD pattern of **Ant‐COF‐OH** (blue dots), Le‐Bail refined profile (red line), difference between experimental and refined pattern (violet line), refined positions of Bragg reflections (blue line symbols), calculated PXRD patterns for simulated AB‐type stack (deep blue line) and AA‐type stack (green line) of **Ant‐COF‐OH**. Calculated structure of **Ant‐COF‐OH**: b) view along the c‐axis, single layer shown, c) view along the a‐axis.

Most of the simulated PXRD patterns for the stable **Ant‐COF‐H** and **Ant‐COF‐OH** topologies (Figures S3 and S4) display characteristic peaks at approximately 5°. The presence of such peaks, corresponding to the 100 or 010 reflections, is expected, as the lattice vectors of the proposed models fall within the 15–25 Å range, dictated by the length of the DPA‐motif. Notably, the experimental PXRD patterns of **Ant‐COF‐H** and **Ant‐COF‐OH** lack peaks in this region (Figures 5a and 6a). This distinctive feature was used as a key criterion for structural elucidation. Among the DFT‐simulated structures, the absence of reflections around 5° was observed only for the AB stacking mode of the *p‐hcb* topology, characterized by a layer shift along the b direction (Figures S4 and S5). In this case, the absence of the 010 reflection is attributed to the structural factors. Finally, the best agreement between the simulated and experimental PXRD patterns was obtained for the *p‐hcb* topology with AB stacking and a layer shift along the b direction in both **Ant‐COF‐H** and **Ant‐COF‐OH** (Figures 5a and 6a). Le‐Bail refinement of the experimental PXRD data yielded refined lattice parameters consistent with the proposed structural model (Table S2).

Nitrogen sorption measurements at 77 K revealed a Brunauer–Emmett–Teller (BET) surface area of 363 m^2^ g^−1^ for **Ant‐COF‐H** and 256 m^2^ g^−1^ for **Ant‐COF‐OH** (Figure 3d). The pore size distribution was evaluated using non‐local density functional theory (NLDFT). The analysis did not allow for unambiguous identification of pore sizes. Therefore, theoretical pore size distributions were calculated using both AA and AB stacking models for **Ant‐COF‐H** and **Ant‐COF‐OH**, employing nitrogen as the probe molecule (*d* = 1.67 Å, Section S1). In all examined cases, the N_2_ probe revealed no accessible channels (Table S3). This observation indicates that the anthracene cores partially fill the COF pores restricting their accessibility (Figure S6), in agreement with the DFT modelling. Consequently, the observed low porosity of the analysed COFs, as well as the pore size distribution evaluated by gas sorption measurements, may be attributed to structural defects and the morphology of the materials, rather than the pores themselves.

**FIGURE 7:**
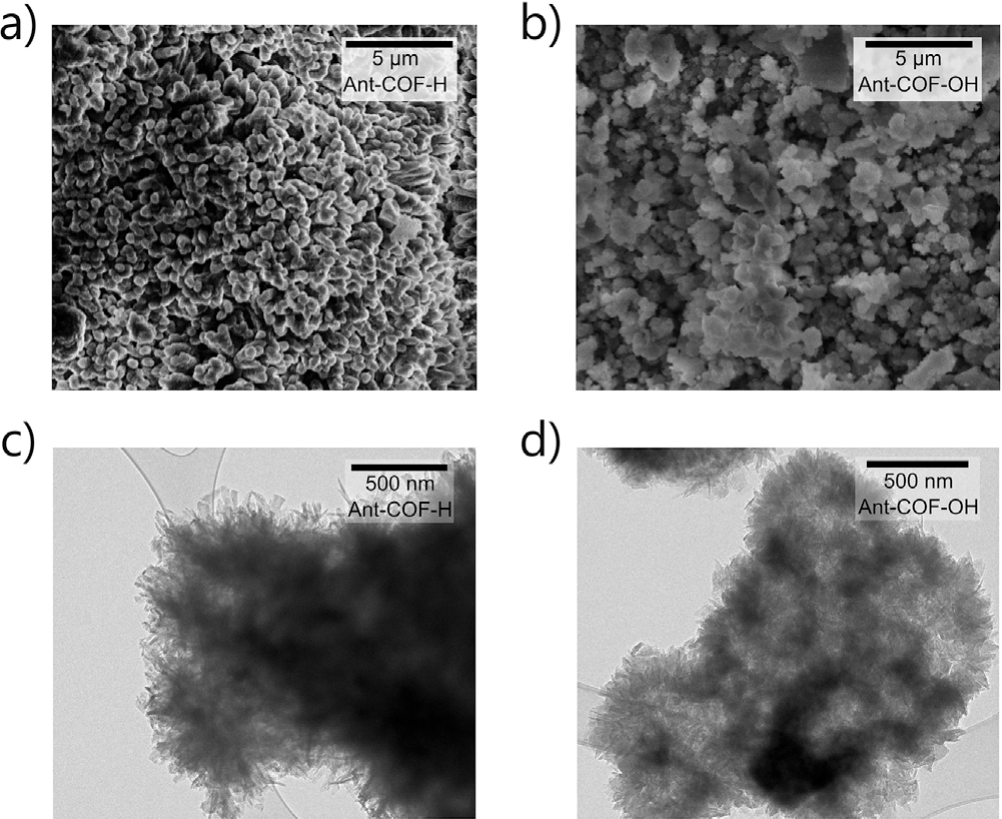
SEM images (a, b) and TEM images (c, d) of **Ant‐COF‐H** and **Ant‐COF‐OH**.

Scanning electron microscopy (SEM) and transmission electron microscopy (TEM) reveal that both **Ant‐COF‐H** and **Ant‐COF‐OH** exhibit similar morphologies characterized by the presence of pillar‐like structures (Figures 7 and S8). The observed porosity of both materials is attributed to adsorption on the irregular surfaces of the COF particles. Additionally, thermal analysis showed that both materials possess comparable thermal stability, with decomposition temperatures around 200°C (Figure S9).

### 2.3 Photophysical Properties of Materials

To assess the upconversion feasibility of the synthesized **Ant‐COF‐H** and **Ant‐COF‐OH**, we investigated their photophysical properties using PdOEP as a model photosensitizer. Triplet–triplet annihilation upconversion (TTA‐UC) imposes two key energetic constraints. First, the energy of the annihilator's first singlet state *E*(S_1_^A^) must be lower than twice the energy of its first triplet state *E*(T_1_^A^), following the relation 2 *E*(T_1_^A^) > *E*(S_1_^A^). This energy surplus permits the generation of a singlet state *via* the annihilation of two triplet states. Second, efficient TTET from the sensitizer to the annihilator requires the sensitizer triplet (T_1_^S^) to be higher in energy than that of the annihilator (T_1_^A^), *E*(T_1_^S^) > *E*(T_1_^A^). This guarantees downhill, directional energy transfer between two species.

**FIGURE 8:**
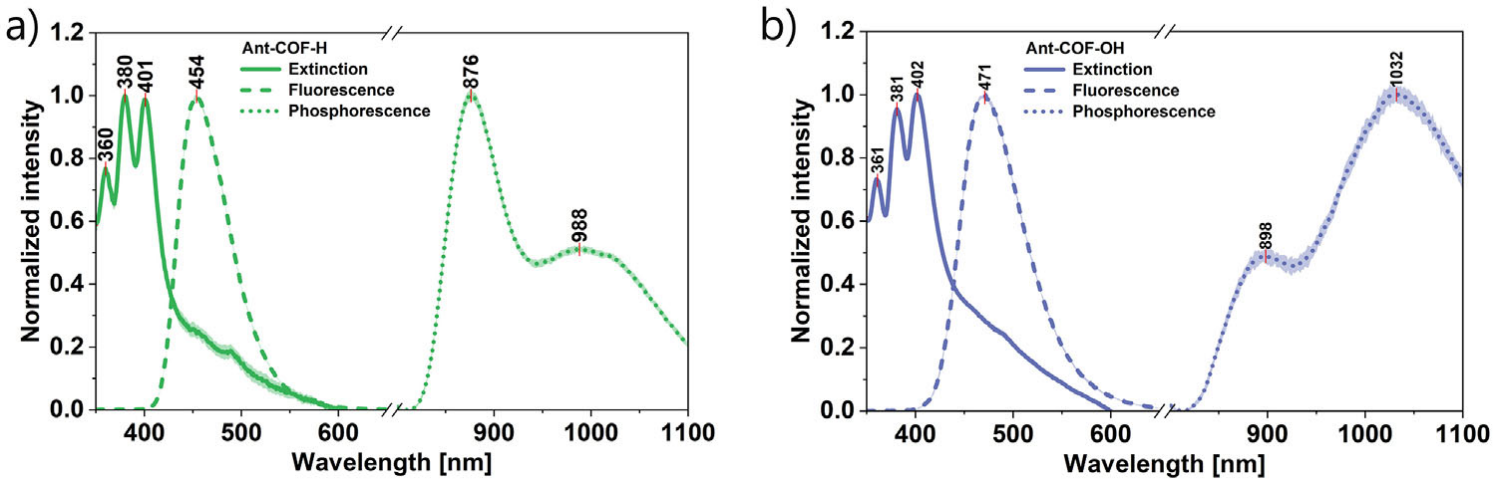
Spectroscopic characteristics in 1,2‐dibromoethane: extinction, fluorescence and phosphorescence spectra of a) **Ant‐COF‐H** and b) **Ant‐COF‐OH**.

To determine whether the first condition is satisfied for **Ant‐COF‐H** and **Ant‐COF‐OH**, the energies of their lowest singlet and triplet states were evaluated spectroscopically. For this purpose, absorption and emission spectra of COFs dispersed in deoxygenated 1,2‐dibromoethane were measured where the heavy‐atom effect of bromine promotes intersystem crossing from S_1_ to T_1_, and thereby enhances phosphorescence emission (Figure 8). Additionally, extinction, fluorescence, and fluorescence lifetime were measured in toluene, the solvent chosen for subsequent light upconversion experiments (Figures S10–S13). To further investigate solvent influence on emission, fluorescence spectra and lifetimes were also recorded in the solid state. For the PdOEP sensitizer, absorbance and luminescence spectra were recorded in deoxygenated toluene (Figures S13 and S21).

The energies of the electronic states of **Ant‐COF‐H**, **Ant‐COF‐OH**, and PdOEP are summarized in Table 1. A comparison of the S_1_^S^ and T_1_^A^ state energetics reveals an energy offset around 0.1 eV for both COFs investigated that satisfies the triplet–triplet energy reserve required for TTA. Furthermore, T_1_^S^ energy of PdOEP lies above the energy of triplet states of the COFs (T_1_^A^), enabling their population through TTET from the porphyrin donor. This fulfills the second key energetic requirement for upconversion.

**TABLE 1:** Photophysical parameters of **Ant‐COF‐H**, **Ant‐COF‐OH**, and PdOEP.

	Absorption [eV]	Fluo. *E*(S_1_) [eV]	Phos. *E*(T_1_) [eV]	TTE reserve 2 *E*(T_1_)−*E*(S_1_) [eV]	Fluo. QY *Φ_F_* [%]	Fluo. LT *τ_F_* [ns] (*A^F^*)
**Ant‐COF‐H**	3.44,3.26,3.09^a^ 3.46,3.29,3.13^b^	2.73^a^ 2.79^b^ 2.72^c^	1.42^a^	0.11^a^	43.16^b^ 15.57^c^	3.18 (1.24)^b^ 0.768 (0.32)^c^ 4.280 (0.40)^c^
**Ant‐COF‐OH**	3.43,3.25,3.08^a^ 3.46,3.28,3.13^b^	2.63^a^ 2.69^b^ 2.56^c^	1.38^a^	0.13^a^	32.29^b^ 16.30^c^	3.50 (1.24)^b^ 0.946 (0.76)^c^ 4.149 (0.33)^c^
**PdOEP**	3.14,2.42,2.27^b^	2.24^b^	1.86^b^	—	—	—

Fluo.—fluorescence; Phos.—phosphorescence; Fluo. QY—fluorescence quantum yield, Fluo. LT—fluorescence lifetime with amplitude (*A^F^*)

^a^ 1,2‐dibromoethane

^b^ toluene

^c^ solid state [Correction added on 26 February 2026, after first online publication: Textual errors have been updated in this version.]

Noticeably, the emission of **Ant‐COF‐H** and **Ant‐COF‐OH** reveals a bathochromic shift with respect to the parent DPA [41, 44]: 0.29 eV for **Ant‐COF‐H** and 0.39 eV for **Ant‐COF‐OH**. This shift, together with the diminished vibronic structure of COF emission bands, suggests the presence of significant interactions between anthracene units within the COF matrix. Further examination of the emissive properties, however, revealed quantum yields *Φ_F_* of 43% and 36% for **Ant‐COF‐H** and **Ant‐COF‐OH**, respectively, when measured in toluene suspensions and excited at 380 nm. Transitioning from the suspension to the purely solid‐state regime resulted in a reduction of the emission quantum yields *Φ_F_* to approximately 16% for both COFs, with additional broadening of the spectra followed by red‐shift (Figure S11). These results indicate that although the anthracene cores interact with each other, this interaction does not lead to emission quenching in the suspension system, but it does in the pure solid state. Therefore, material properties revealed in suspension regime can be beneficial for the upconversion process, where COF particles must both support excited triplet state migration and emit light from the generated singlet states.

Time‐resolved fluorescence measurements reveal that in toluene suspensions the COFs exhibit a clean mono‐exponential decay (3.18 ns for **Ant‐COF‐H** and 3.50 ns for **Ant‐COF‐OH**), comparable to monomeric DPA [41], indicating a DPA‐like emissive state. In contrast, emissions from solid‐state samples are fitted with bi‐exponential functions comprising a fast sub‐nanosecond component attributed to traps or aggregate‐assisted quenching, along with a slower ∼4 ns component that reflects the intrinsic DPA S_1_ lifetime (Figure S12). The radiative timescale remains essentially DPA‐like in solvent, and reveals characteristics of aggregates in solid state, consistent with observed reduction in emission quantum yields *Φ_F_* when transitioning from suspension to solid state. Overall, the presented physicochemical characteristics of both COFs show that these materials are suitable for the role of emitter in the upconversion process.

The **Ant‐COF‐H** and **Ant‐COF‐OH** functionality as emitters has been tested in degassed toluene in the presence of PdOEP as a photosensitizer. Because the upconversion performance depends on the concentrations of sensitizer and emitter, an initial screening of the suspension composition was carried out by varying the concentrations. Then upconversion quantum yields and power‐law exponents (n) were extracted from double logarithmic plots of the integrated PdOEP fluorescence and phosphorescence intensities (Table S4, Figures S16 and S17). Ultimately, systems containing **Ant‐COF‐H** and PdOEP at concentrations of 0.032 mM and 0.017 mM, respectively, and **Ant‐COF‐OH** and PdOEP at concentrations of 0.037 mM and 0.017 mM, respectively, were selected. For comparison, the solution of parent DPA and PdOEP at analogous concentrations was used as a reference system (Table S4, Figure S18).

**FIGURE 9:**
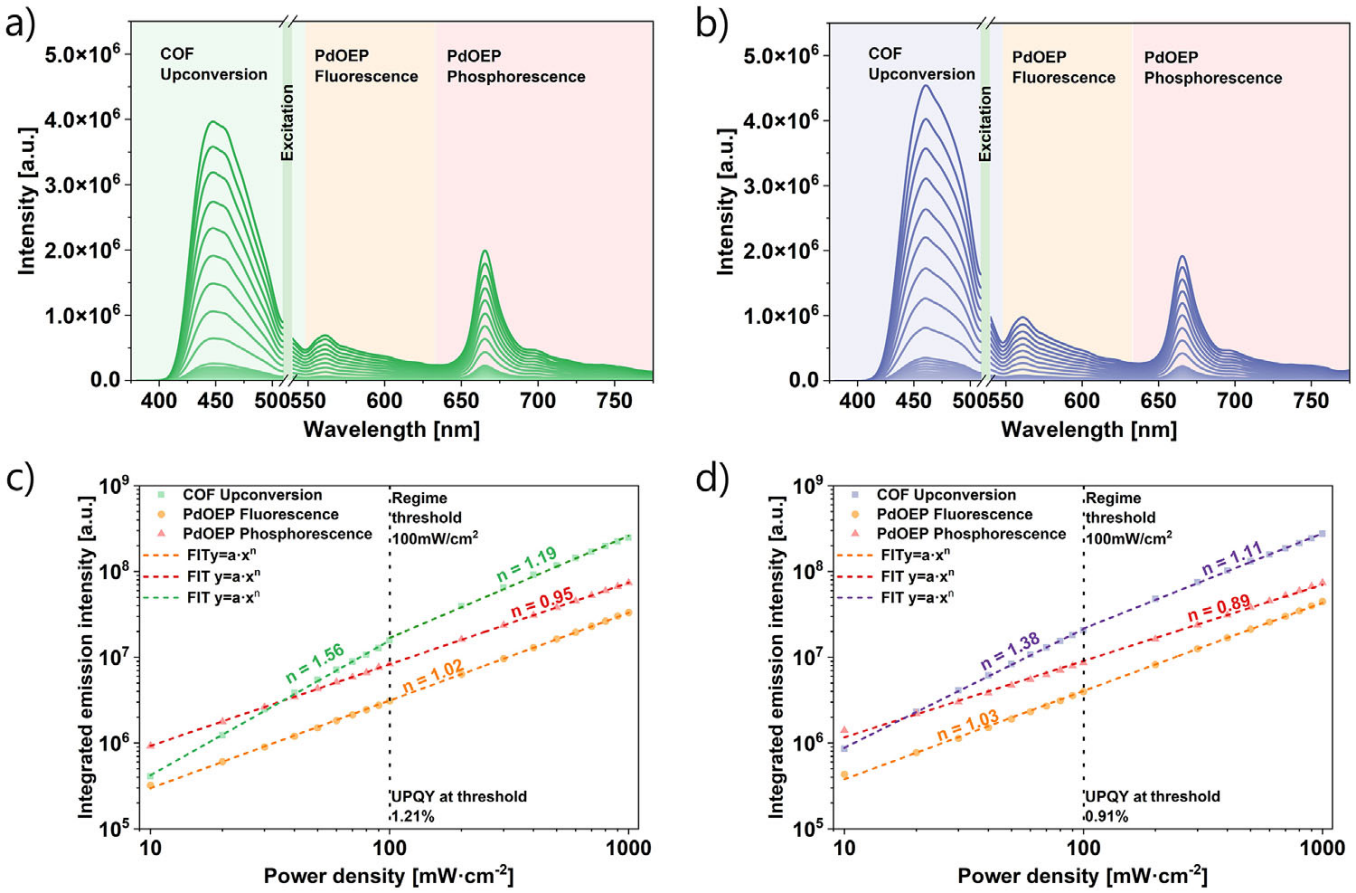
TTA upconversion luminescence spectra recorded for excitations power densities of 10–1000 mW cm^−2^ for **Ant‐COF‐H** (a) and **Ant‐COF‐OH** (b) using PdOEP as a sensitizer. Double logarithmic plots of integrated emission intensities as a function of the excitation power density: upconverted emission of **Ant‐COF‐H** (c) and **Ant‐COF‐OH** (d), together with PdOEP fluorescence and phosphorescence, and linear fits of different slopes n indicate distinguishable operation regimes of the upconverting systems.

The upconversion performance of **Ant‐COF‐H** and **Ant‐COF‐OH** was monitored by recording emission spectra in an integrated sphere setup under excitation power densities ranging from 10–1000 mW cm^−2^ (Figure 9a and b). For both COFs, the emission spectra consist of four components: the COF emission 400–520 nm with a maximum at 454 nm for **Ant‐COF‐H** and at 471 nm for **Ant‐COF‐OH**, a residual signal from scattered excitation at 523 ± 3 nm, residual PdOEP fluorescence at 550–630 nm, and residual PdOEP phosphorescence at 630–775 nm (Figures 9a,b, S16 and S18). The dependence of the upconverted emission intensity on excitation power density, plotted on a doubly logarithmic scale, reveals a clear deviation from the quadratic behavior expected at low power densities (Figures 9c,d, S17 and S18). On log–log plots, many diffusion‐limited solution systems show a relatively sharp crossover from a quadratic regime (n ≈ 2) at low excitation densities to a linear regime (n ≈ 1) once the triplet population approaches steady‐state saturation. In contrast, for **Ant‐COF‐H** and **Ant‐COF‐OH** the power dependence below ∼100 mW cm^−^^2^ is clearly sub‐quadratic, with apparent exponents of ∼1.6 and ∼1.4, respectively, indicating a broad intermediate (“mixed‐kinetics”) region rather than a distinct n = 2 plateau. Sub‐quadratic scaling has previously been attributed to the presence of semisolid and polymeric emitters [45, 46, 47, 48]. However, in the present study the same intermediate scaling is also observed for the molecular PdOEP/DPA reference under identical measurement conditions (Table S4, Figure S18), demonstrating that the behavior is not intrinsically linked to the polymeric/solid form of the acceptor. Consistent with this, steady‐state kinetic modeling reproduces the intermediate slope and attributes it to competition between first‐order triplet loss/transfer steps and second‐order TTA under incomplete triplet‐state saturation (Section S1, Table S8, Figure S22). At higher excitation densities the apparent exponent approaches unity, and the boundary of the “linear” regime was therefore operationally defined by the best‐fit range yielding n ≈ 1 (see Section S1).

Time‐resolved emission spectroscopy was performed for both described systems as a function of excitation power. Emission was collected at 450 nm, and excitation was set to 523 nm using a pulsed laser system (Section S1). For all scanned power densities, a single‐exponential rise transitioning to bi‐exponential decay of a signal was observed (Table 2, Figure S19). The decay times of both **Ant‐COF‐H** and **Ant‐COF‐OH** fall within the timescale expected for internal TTA. Two temporal components were obtained *τ_1_* ≈ 65 µs, *τ_2_* ≈ 20 µs and *τ_1_* ≈ 50 µs, *τ_2_* ≈ 20 µs for each COF respectively. Importantly, the absence of a longer decay component (> 150 µs) excludes diffusion‐dependent processes in the relaxation pathways, demonstrating that the kinetics are not limited by external TTA [28] and highlighting the intra‐framework nature of the process. The rise time *τ_3_* ≈ 6.5 µs (for both COFs), on the other hand, is a convolution of processes required to populate the annihilator triplet state, including sensitizer absorption, EET, and TTET. Because TTET in sensitizer solution‐based system is substantially slower than the other phenomena in this sequence, it is rate‐limiting and thus dominates the overall rise. Moreover, the rise time is significantly shorter than the reported lifetime of the PdOEP triplet state, which ranges from 200 to 300 µs [49, 50, 51]. This showcases a small‐molecule diffusion nature of yet an efficient triplet energy transfer process, which outcompetes the sensitizer's intrinsic radiative decay (parasitic phosphorescence).

**TABLE 2:** Measured photon upconversion yields *Φ_UC_*, calculated TTET quantum yields *Φ_TTET_*, and annihilation quantum yields as a product of *Φ_EET_* · *Φ_TTA_*, averaged over excitation power densities of 100–1000 mW cm^−2^, and extracted lifetimes of upconverted emission decays (*τ_1_* and *τ_2_*), and rise (*τ_3_*) together with their amplitudes *A* averaged over excitation power densities of 2–200 mW cm^−2^.

	*Φ_UC_* [%]	*Φ_TTET_* [%]	*Φ_EET_* · *Φ_TTA_* [%]	*τ_1_* [µs] (*A_1_*)	*τ_2_* [µs] (*A_2_*)	*τ_3_* [µs] (*A_3_*)
**Ant‐COF‐H**	1.78 ± 0.25	44.52 ± 1.36	18.87 ± 2.99	69.43 ± 4.38 (0.49 ± 0.30)	24.31 ± 2.49 (1.39 ± 0.27)	6.82 ± 1.69 (−2.06 ± 0.12)
**Ant‐COF‐OH**	1.18 ± 0.13	67.18 ± 0.99	9.74 ± 1.18	44.13 ± 4.38 (0.66 ± 0.38)	18.48 ± 2.82 (1.54 ± 0.28)	6.39 ± 1.45 (−2.40 ± 0.37)

The upconversion quantum yield (*Φ_UC_*) was calculated in an absolute manner by comparing the number of photons absorbed by the sensitizer to the number of photons emitted by the annihilator and, therefore, limiting the maximal theoretical yield to 50% in a bimolecular TTA system. We recorded *Φ_UC_* of ∼1.8% and ∼1.2% at the power density threshold for **Ant‐COF‐H** and **Ant‐COF‐OH**, respectively. When the upconversion pathway is as described in Figure 1, then overall *Φ_UC_* can be expressed as a function of efficiencies of subprocesses following the Equation (1):

(1) $$\Phi_{UC}=\;\;\frac{1}{2}\cdot \Phi_{ISC}\cdot \Phi_{TTET}\cdot \Phi_{EET}\cdot \Phi_{TTA}\cdot \Phi_{F_{A}\;}$$

where *Φ_ISC_* is the intersystem crossing efficiency, *Φ_TTET_* is the triplet‐triplet energy transfer efficiency, *Φ_EET_* stands for the internal exciton energy transfer efficiency, *Φ_TTA_* is the overall triplet‐triplet annihilation efficiency, and *Φ_F_* is the fluorescence quantum yield of the annihilator sample measured at the same conditions but excited directly to its S_1_ state. The ½ factor accounts for bimolecular nature of the process.

The *Φ_TTET_* is derived for each power density by comparing the phosphorescence quantum yield of PdOEP in upconverting systems with that of a reference solution containing solely porphyrin at the same concentration and under the same conditions:

(2) $$\Phi_{TTET}=\;\;\frac{{\Phi_{P_{S}\;}}^{\prime }-\Phi_{P_{S}\;}}{{\Phi_{P_{S}\;}}^{\prime }}$$

*Φ_PS_′* is phosphorescence quantum yield of the sensitizer in reference sample and *Φ_PS_* is the same quantity in the upconverting system (Table S4). Although, the triplet–triplet annihilation efficiency *Φ_TTA_* cannot be determined independently, the product *Φ_EET_* · *Φ_TTA_* can be calculated from Equation (1). This calculation assumes unit intersystem crossing efficiency (*Φ_ISC_*) [52] and utilizes the annihilator's fluorescence quantum yield (*Φ_F_*) previously measured for toluene‐based samples (Table 1).

TTET is strongly concentration‐dependent. In DPA‐based frameworks, optimal incorporation of annihilator units can yield *Φ_TTET_* values of ∼80% in porous solids [19] and up to ∼99% in well‐mixed molecular solutions [53], whereas our reference DPA/PdOEP solution exhibits a TTET efficiency of 72% (Table S5). By contrast, in dispersions the local sensitizer–annihilator environment often deviates from the bulk average, thereby limiting encounter probabilities. Nevertheless, our determined *Φ_TTET_* values of ∼45% for **Ant‐COF‐H** and ∼70% for **Ant‐COF‐OH** (Table 2) are only modestly below these benchmarks, indicating efficient sensitizer–acceptor contact despite heterogeneity. Notably, the annihilation efficiency (*Φ_EET_* · *Φ_TTA_*) of ∼19% for **Ant‐COF‐H** and ∼10% for **Ant‐COF‐OH** exceeds that of the reference DPA/PdOEP solution (∼4%; Table S5). This improvement is reflected in the overall higher upconversion quantum yields observed for the COF‐based systems (Table 2) compared with the solution reference (1.24%, Table S5). These results demonstrate that COF‐based upconverters can outperform solution systems due to more efficient exciton energy transfer and triplet–triplet annihilation, enabled by diffusion‐independent energy transport within the framework.

Finally, the annihilation efficiencies (*Φ_EET_* · *Φ_TTA_*) can be directly correlated with the structural features of **Ant‐COF‐H** and **Ant‐COF‐OH**. Both processes, EET and TTA, rely on the Dexter energy transfer mechanism, which requires short‐range interactions mediated by orbital overlap between energy‐exchanging sites [54]. Structural models reveal that the AB‐type stacking in the studied COFs offsets DPA‐moieties, positioning the anthracene cores adjacent to piperazine linkers in neighboring layers (Figures 5b, 6b, and S6). This alignment of anthracene cores restricts the probability of energy transfer and triplet–triplet annihilation between them, forming an energy loss pathway. Rational control over COF layer alignment, particularly the relative orientation and interlayer spacing of embedded emitters, could therefore enhance overall upconversion efficiency. Such control might be achieved through the introduction of substituents that stabilize the AA‐stacked configuration relative to the AB stacking mode. The relationship between COF stacking and upconversion performance will be explored in future studies.

## 3 Conclusion

Aminal‐linked covalent organic frameworks incorporating anthracene units, **Ant‐COF‐H** and **Ant‐COF‐OH**, were successfully synthesized in high yield (∼90%). PXRD analysis confirmed their high crystallinity. Density functional theory (DFT) geometry optimization of the periodic systems provided plausible structural models, identifying two‐dimensional polymers with *p‐hcb* topology and AB stacking. The incorporation of nonplanar piperazine units, together with interrupted intralayer π‐conjugation, afforded high photoluminescence quantum yields of up to 40%. Upon sensitization with a palladium porphyrin complex, both **Ant‐COF‐H** and **Ant‐COF‐OH** exhibited upconverted emission with quantum yields of up to 1.8%, with system saturation observed at an excitation power density of 100 mW cm^−2^. The dependence of the upconverted emission on excitation power revealed a gradual transition from the non‐saturated to the saturated regime, rather than the typical sharp slope change (from n = 2 to n = 1), reflecting mixed orders of TTET and TTA kinetics. Time‐resolved emission spectroscopy showed short rise times of approximately 10 µs, associated with fast TTET kinetics and confirming the absence of diffusion‐limited processes. The extracted TTA efficiencies were 10% and 19% for **Ant‐COF‐H** and **Ant‐COF‐OH**, respectively, outperforming the reference solution‐based system, despite restricted Dexter‐type energy transfer due to the offset alignment of anthracene units. These findings demonstrate that piperazine‐linked aminal COFs can function as solid‐state emitters in the upconversion process, establishing a new platform for photon energy conversion.

## Conflicts of Interest

The authors declare no conflicts of interest.

## Supporting information




**Supporting File 1**: The authors have cited additional references within the Supporting Information.

**Supporting File 2**: anie71574‐sup‐0002‐Data.py.

## Data Availability

This research was funded in whole or in part by National Science Centre, Poland grant no. 2022/47/D/ST5/01375. The datasets generated and analyzed during the current study are available in the RepOD repository at https://doi.org/10.18150/GN7ZRM.
