# Supplementary material for: Aminal‐linked Covalent Organic Frameworks for Light Energy Upconversion
Source: Angew Chem Int Ed Engl. 2026 Feb 23;65(14):e22521. doi: 10.1002/anie.202522521 (PMC13023729; doi:10.1002/anie.202522521)
Supplement: Supplementary file 1 — Supporting File 1: The authors have cited additional references within the Supporting Information. [file ANIE-65-e22521-s001.pdf]

Supporting information for

Aminal-linked Covalent Organic Frameworks for Light Energy Upconversion

Mateusz Brzeziński,[a] Agata Tyszka-Gumkowska,[b] Aleksander Gorski,[a] Marek J. Potrzebowski,[c] Tomasz Polczyk,[d] Wojciech Wegner,[b] Sylwester Gawinkowski,*[a] Jakub Ostapko*[b]

a) Institute of Physical Chemistry, Polish Academy of Sciences, Marcina Kasprzaka 44/52, 01-224 Warsaw, Poland
b) Centre of Excellence ENSEMBLE3 Sp. z o.o., Wólczyńska 133, 01-919 Warsaw, Poland
c) Centre of Molecular and Macromolecular Studies Polish Academy of Sciences, Łódź, Poland
d) Faculty of Chemistry, Jagiellonian University, Gołębia 24, 31-007 Kraków, Poland

Table of contents

1. **General Remarks** S1
2. **Synthesis** S13
3. **COF synthesis optimization** S16
4. **PXRD** S17
5. **Structure elucidation** S18
6. **Infrared spectra** S23
7. **TEM** S23
8. **DSC-TGA** S24
9. **Spectroscopy** S25
10. **Concentration screening for the upconversion** S28
11. **Cartesian atomic coordinates** S33
12. **Kinetic model parameters** S38
13. **Literature** S43

1. General Remarks

Methodology and chemical handling. All solvents and reagents were obtained from common suppliers and used as received. Reaction required anhydrous and anaerobic conditions were performed in oven-dried glassware under an inert atmosphere of N_2 by means of standard Schlenk line and glovebox (GS Systemtechnik and MBraun) techniques. If needed, solvents were dried and stored with molecular sieves (4 Å). Flash column chromatography was performed on silica gel (230–400 mesh); thin-layer chromatography (TLC) was carried out on aluminum sheets coated with SiO_2-60 F254 obtained from Merck; visualization with a UV lamp (254 or 366 nm). All products were dried under high vacuum (ca. 10^{-2} mbar) before analytical characterization.

Nuclear magnetic resonance (NMR) spectra were acquired on the Bruker AVANCE II instrument (300 MHz or 500 MHz) and Agilent DD2 400 MHz. Chemical shifts are reported in parts per million (ppm) and referenced to solvent residue peak. The splitting pattern of multiplets is described by abbreviations (s – singlet, d – doublet, t – triplet, q – quartet, dd – doublet of doublets, m – multiplet, c – covered signal, b – broad peak). Coupling constants (*J*) values are reported in Hz.

Cross-polarization magic angle spinning (CP MAS) NMR experiments were performed on a 400 MHz Bruker Avance III spectrometer, operating at 400.15 MHz and 100.63 MHz for ^{1}H and ^{13}C respectively. equipped with a Broad Band (^{15}N-^{31}P) 4 mm MAS probe head. A sample of U-^{13}C, ^{15}N-labeled histidine hydrochloride was used to set the Hartmann–Hahn condition. The ^{13}C CP MAS spectra were recorded with a proton 90° pulse length of 4 µs, 2 ms contact time, 6s repetition time and SPINAL64 decoupling (83 kHz amplitude). Powdered COF samples were packed into 4 mm ZrO_2 rotor and spun at a spinning rate of 7 kHz and 8 kHz. The 2 K or 4 K transients were acquired, FIDs were accumulated using time domain size of 3.6 K data points. Adamantane (resonances at 38.48 and 29.46 ppm) was used as a secondary ^{13}C chemical-shift reference from external tetramethylsilane (TMS). The NMR data were processed using Bruker Topspin software version 3.5.

High-resolution mass spectrometry (HRMS) measurements were performed using Synapt G2-Si mass spectrometer (Waters).

Elemental Analysis (EA) was obtained on Vario EL Cube (Elementar) apparatus.

Differential Scanning Calorimetry – Thermogravimetric Analysis (TGA) was performed on the SDT Q600 (TA Instruments) under nitrogen (50 L min^{-1}) with a 10°C min^{-1} temperature ramp.

Powder X-Ray Diffraction (PXRD) was performed using a Panalytical Empyrean diffractometer equipped with a Cu Kα radiation source (λ = 1.5406 Å) and operated in Bragg-Brenatano geometry. The measurement range was set to 1.8–40° 2θ, with a step size of 0.039° for synthesis optimization and a step size of 0.014° for synthesis under optimized conditions, with total acquisition times of 1 hour and 8 hours, respectively. The X-ray tube was operated at 45 kV and 40 mA. A programmable divergence slit (1/16°) and a fixed anti-scatter slit (1/8°) were employed to optimize the beam geometry and reduce background noise.

The FullProf 5.20 package with EdPCR 2.0 was used to perform the Le Bail fit.[70] The structure obtained from DFT calculations was used as the preliminary structural model. The peak shape was modeled using a pseudo-Voigt function.

Scanning electron microscopy (SEM) was carried using FEI Nova NanoSEM 450 microscope.

Transmission electron microscopy (TEM) investigations were carried out using an FEI Tecnai Osiris transmission electron microscope equipped with an X-FEG Schottky field emitter operated at 200 kV and a high-angle annular dark-field (HAADF) detector. The microscopic data were analyzed using GMS software. Prior to microscopic analysis, the samples were deposited onto lacey carbon films supported on copper grids (400 mesh, Agar Scientific, London, UK).

N_2 adsorption/desorption measurements were performed using a 3P Instruments Micro200 Surface Area and Porosity Analyzer at 77 K. Before analysis, samples (50 mg–100 mg) were degassed at 120 °C for 10 h under dynamic vacuum. Brunauer-Emmett-Teller (BET) method was utilized to calculate the specific surface areas. The relative pressure regimes for the BET analysis were chosen according to the criteria for evaluating BET surface areas for microporous materials.[71] The pore sizes were derived from the sorption curves using the non-local density functional theory model. BET surface areas were further evaluated using the BETSI software, employing a minimum of 10 data points, a minimum correlation coefficient (R^2) of 0.995, and ensuring compliance with Rouquerol criteria 1–4 as well as a maximum relative error of 20%.[57] The BETSI results showed very good agreement with surface areas calculated using the 3P Instruments software.

Continuous Wave (CW) Laser Setup. A 520 nm laser diode (L520A2, Thorlabs) was driven by a temperature-controlled current source (ITC4020, Thorlabs) operated in constant-current mode at 250 mA with the diode temperature regulated at 0 °C. The diode was mounted in a thermoelectric collimation module (LDM38/M, Thorlabs). The collimating lens (Thorlabs, A240TM) was housed in an SM1 lens tube (S1TM08, Thorlabs) directly coupled to the LDM38/M. Excitation power was adjusted using a variable-polarization attenuator composed of two polarizing beamsplitter cubes (PBS202, Thorlabs). One cube was held in a kinematic mount (KM100P, Thorlabs), and the second was installed in a 1″ cube mount (CCM1-4ER, Thorlabs) fixed to a PRM1/M motorized rotation stage (Thorlabs) to vary the relative polarization and thus the transmitted power. The beam was guided along the optical rail by broadband dielectric mirrors (BB1-E03, Thorlabs) and finally focused onto the sample using an N-BK7 lens (LB1779-ML, Thorlabs). Laser power and irradiance at the sample plane were quantified with a power meter (PM100D, Thorlabs) paired with a calibrated Si photodiode sensor (S121C, Thorlabs). The beam area at the sample was defined by the knife-edge (blade) method: a utility-knife blade was mounted on a linear translation stage (XR50P/M, Thorlabs) at the sample position and advanced stepwise across the focused beam while recording transmitted power with the PM100D/S121C. The resulting power-versus-position trace was fit (Gaussian beam assumption) to extract the beam diameter at the measurement plane, from which the spot area was obtained. Power density was reported as the measured power divided by the spot area. Before each measurement, current and temperature setpoints were allowed to stabilize, and the optical path (polarizers, mirrors, and focusing lens) was kept fixed throughout all acquisitions. All components were sourced from Thorlabs (Newton, NJ, USA).

Puls Laser (PL) Setup. A pulsed laser (OPOTEK Radiant 355) operated at a 10 Hz repetition rate and tuned to 523 nm and 5 ns FWHM served as the excitation source. Triggering and timing were provided by a digital delay/pulse generator (DG535, Stanford Research Systems). Pulse energy was adjusted using a variable-polarization attenuator composed of two polarizing beamsplitter cubes (PBS202, Thorlabs). One cube was mounted in a kinematic mount (KM100P, Thorlabs), and the second cube was installed in a 1″ cube mount (CCM1-4ER, Thorlabs) fixed to a PRM1/M motorized rotation stage (Thorlabs) to vary the relative polarization and thus the transmitted pulse energy. The beam was guided by broadband dielectric mirrors (BB1-E03, Thorlabs) and focused onto the sample with an N-BK7 lens (LB1779-ML, Thorlabs). Pulse energy and fluence at the sample plane were measured with a power/energy meter console (PM100D, Thorlabs) paired with a pyroelectric energy sensor (ES308C, Thorlabs). For fluence determination, the beam area at the sample was defined by the knife-edge (blade) method: a utility-knife blade was mounted on a linear translation stage (XR50P/M, Thorlabs) at the sample position and advanced stepwise across the focused beam while recording shot-to-shot energy with the PM100D/ES308C. The resulting energy-versus-position trace was fit under a Gaussian-beam assumption to extract the beam diameter at the measurement plane, from which the spot area was obtained; fluence was reported as the measured pulse energy divided by this area. Prior to data collection, the repetition rate and trigger outputs were verified for stability, and the optical path (polarizers, mirrors, and focusing lens) was kept fixed for all measurements. All optical components were sourced from Thorlabs (Newton, NJ, USA) unless otherwise specified. The setup was equipped with a Hamamatsu R955 photomultiplier and a Yokogawa DL9140 fast oscilloscope for time-resolved measurements.

Preparation of Solutions and Suspensions. All solutions of building blocks compounds and suspensions of polymeric COFs were prepared in spectroscopic purity grade solvents (Sigma Aldrich, Supelco, Uvasol series). The solvents used were freshly opened. To further purify and dry the solvents and remove dissolved oxygen, they were transferred into 20 mL vials, to which approximately 10–20 beads of 4 Å molecular sieves were added. The solvents were then transferred to an extractor, the bottom of which was also filled with identical molecular sieves (approximately 50 g). Each solvent was degassed individually by applying a vacuum until bubbling ceased, followed by purging the chamber with nitrogen. This vacuum-purge cycle was repeated five times, after which the solvents were left for an additional 24 hours. The degassing process (five additional cycles) was then repeated the following day. The samples were then mixed using a vortex mixer for at least 60 s, followed by ultrasonic treatment in a bath sonicator for 10 min without heating.

For O_2 free measurements, samples were degassed by five freeze–pump–thaw (FPT) cycles using a liquid-nitrogen bath and an oil-sealed rotary vane pump. Samples were contained either in a quartz cuvette (Hellma, HL111-10-40) that was custom glass blown (flame-fused) to a 20 mL Schlenk tube, or in 2 mL glass ampoules connected *via* a Schlenk valve. Each FPT cycle consisted of freezing the sample to a solid, evacuating the headspace to $< 10^{-3}$ mbar, isolating the vessel, and allowing the sample to thaw to room temperature. After the fifth cycle, the vessels with frozen sample were kept under dynamic vacuum at $< 10^{-3}$ mbar and flame-sealed prior to measurement to form a permanent seal. Cuvettes equipped with Schlenk tube were evacuated to $< 10^{-3}$ mbar and then screw-sealed using a PTFE-built-in stop cork. All manipulations were carried out using standard Schlenk techniques. Before all spectroscopic

measurements, the suspensions were subjected to 1 min gentle stirring using a vortex to prevent sedimentation of the powder at the bottom of the container.

Absorbance Measurements of Solutions and Suspensions. Absorbance measurements were performed using quartz cuvettes (Hellma, HL110-1-40) with an optical path length of 0.1 cm and a Shimadzu UV-Vis 2700 spectrophotometer. The baseline was established by filling both cuvettes with pure solvent and recording a spectrum in the 200–800 nm range. During the actual measurements, a cuvette filled with pure solvent was used as a reference, and spectra were recorded in the 300–800 nm range, with each measurement repeated three times. All measurements were conducted with the excitation and emission monochromator slit widths set to 5 nm, with an accumulation time of 1 nm/0.1 s and a wavelength step size of 0.5 nm. No step correction was applied during the lamp-switching which wavelengths were set at 290 nm and 1100 nm. Data acquisition was performed using the UV Probe ver. 2.43 software.

Absorbance Data Processing for Solutions and Suspensions. The data, recorded in ASCII format as absorbance vs. wavelength, were imported into OriginPro 2024b. For each sample, three consecutive spectra were collected. A relevant wavelength range was selected using a masking function to exclude solvent absorbance (lower wavelength range < 300 nm / < 350 nm). The spectra were then averaged to obtain a mean spectrum without further smoothing. The processed spectra were used to generate final plots without further modifications.

Luminescence Measurements of Solution and Suspensions in Dibromoethane. Luminescence measurements were performed using quartz cuvettes (Hellma, HL111-10-40) with an optical path length of 1 cm and a Varian Cary Eclipse fluorescence spectrophotometer. Excitation wavelengths ranged from 335 nm to 395 nm, with a step size of 20 nm. Emission data were collected in the 300–1100 nm range. During each measurement, the photomultiplier voltage was adjusted to ensure that the emission peak fell within the range of 200–800 counts. If this was not achievable due to weak luminescence, the voltage was set to its maximum value. All measurements were conducted with the excitation and emission monochromator slit widths set to 5 nm (phosphorescence) and 2.5 nm (fluorescence), with an accumulation time of 1 nm/0.1 s or at a slower rate, with a wavelength step size of 1 nm. Data acquisition was performed using the Scan ver. 1.1(132).

Luminescence Data Processing for Solutions and Suspensions in Dibromoethane. The data, recorded in ASCII format as counts vs. wavelength, were imported into OriginPro 2024b for initial visualization. Spectra for each excitation wavelength were plotted, and a masking function was applied to manually remove regions outside the region of interest and the excitation lamp wavelength. Data for all excitation wavelengths were normalized 0-1 intensity scale. The processed spectra were used to generate final plots without further modifications.

Luminescence Measurements of Solutions and Suspensions in Toluene. Measurements were performed using a Horiba Fluorolog modular spectrofluorometer (1997) equipped with an integrating sphere QuantaPhi-2. The experiments were conducted in Integrating Sphere (IS) mode. The solutions/suspensions were placed inside 2 mL sealed glass ampules. The prepared sample was suspended at the center point of the integrating sphere. The incident light port was directed from the front, directly on the sample, while the exit port was connected to the side part of the sphere, forming a 90-degree angle

between the two ports. All measurements were performed with an accumulation time of 1 nm/1 s and a wavelength step size of 1 nm. Data were collected from the main detector (within its linear range, up to 1 million counts) and the reference detector. The excitation and emission monochromator slit widths were set to 1.5 nm. Samples were excited with a 380 nm line, and their luminescence were collected in the range of 400–720 nm. No filter was used for this type of measurement. Reference measurements were performed identically by replacing the samples with fine-ground $BaSO_4$ powder. Data acquisition was carried out using the FluorEssence ver. 3.51.20 with Origin ver. 8.1090 software.

Scattering Measurements of Solutions and Suspensions in Toluene. Measurements were performed using a Horiba Fluorolog modular spectrofluorometer (1997) equipped with an integrating sphere QuantaPhi-2. The experiments were conducted in Integrating Sphere (IS) mode. The solutions/suspensions were placed inside 2 mL sealed glass ampules. The prepared sample was suspended at the center point of the integrating sphere. The incident light port was directed from the front, directly on the sample, while the exit port was connected to the side part of the sphere, forming a 90-degree angle between the two ports. All measurements were performed with an accumulation time of 1 nm/1 s and a wavelength step size of 0.5 nm. Data were collected from the main detector (within its linear range, up to 1 million counts) and the reference detector. The excitation and emission monochromator slit widths were set to 1.5 nm. Samples were excited with a 380 nm line and their luminescences were collected in the range of 400–720 nm. Neutral density filters (Edmund Industrial Optics) OD1.5 was used. The reference measurements were performed identically by replacing the samples with pure solvent. Data acquisition was carried out using the FluorEssence ver. 3.51.20 with Origin ver. 8.1090 software.

Luminescence and Scattering Data Processing of Solid States/Solutions and Suspensions in Toluene. The data, recorded in .opj format, were processed using OriginPro 2024b. All spectra were multiplied by a correction function for the integrated sphere spectral profile. Scattering measurements were additionally corrected (divided) based on the OD filters' spectral profile and multiplied by Rayleigh scattering scaling factor of 0.7 (solutions and suspensions) and 0.33 (solid states). Data was normalized 0-1 intensity scale. The processed spectra were used to generate final plots without further modifications.

Quantum Yield calculation. The fluorescence quantum yield (Φ_F) was determined absolutely by relating the number of photons emitted by the sample to the number of photons absorbed under the same excitation conditions. By definition:

$$\Phi_F = \frac{N_{em}}{N_{abs}} \qquad \text{(eq. S1)}$$

where N_{em} is the total emitted-photon count and N_{abs} is the total absorbed-photon count.

The total number of photons emitted by the sample and collected by the sphere is:

$$\mathrm{N}_{em} = \int_{\lambda_{em}} C_{em,sam}(\lambda) \cdot k(\lambda)\, \mathrm{d}\lambda \qquad \text{(eq. S2)}$$

where $C_{em,sam}(\lambda)$ is the sample's photon-count spectrum recorded in the emission band with the excitation light rejected and $k(\lambda)$ is the counts-per-photon calibration of the detection channel.

Absorption was obtained from the decrease in the excitation-band scattering inside the sphere by comparing a non-absorbing reference to the sample, both measured under identical excitation:

$$\mathrm{N}_{abs} = \int_{\lambda_{ex}} \frac{C_{scat,ref}(\lambda) - C_{scat,sam}(\lambda)}{j(\lambda)} \cdot k(\lambda) \cdot f_{ex}\, \mathrm{d}\lambda \qquad \text{(eq. S3)}$$

where $C_{scat,ref}(\lambda)$ and $C_{scat,ref}(\lambda)$are the photon-count spectra of the scattered excitation for the reference and the sample, respectively, $j(\lambda)$ is OD filter absorption correction and f_{ex} is Rayleigh scattering scaling factor. Equation 3 for CW laser-excited measurement evolves to:

$$\mathrm{N}_{abs} = \int_{\lambda_{ex}} \frac{C_{scat,ref}(\lambda) - C_{scat,sam}(\lambda)}{j(\lambda)} \cdot k(\lambda) \cdot f_{ex} \cdot \eta_{spot}\, \mathrm{d}\lambda \qquad \text{(eq. S4)}$$

where η_{spot} is a dimensionless correction factor that restricts the absorbed-photon count to first-pass absorption within the directly illuminated laser spot, excluding photons absorbed elsewhere in the sample by the sphere's diffuse field. It is obtained from two geometries – A: direct spot (primary and secondary absorption), and B: sphere-diffuse (secondary absorption only) – as:

$$\eta_{spot} = 1 - \frac{N_{abs}^{(A)}}{N_{abs}^{(B)}} \qquad \text{(eq. S5)}$$

Luminescence Intensity vs Power Density Threshold Estimation. Intensity vs. excitation power density plots were analyzed in OriginPro 2024b using the built-in Allometric model with nonlinear least squares (default settings). For each dataset, the entire power range was fit to obtain a global exponent. To identify the asymptotic regime and estimate the threshold, the lowest-power point was iteratively excluded, the model refit after each exclusion, and the change in exponent monitored. Iterations were terminated once the slope stabilized. The stabilized exponent was reported, and the threshold power density was defined as the lowest power remaining in the converged subset.

Fluorescence Lifetime Measurements for Solution, Suspensions and Solid States. Lifetime measurements were conducted using the Time-Correlated Single Photon Counting (TCSPC) method with quartz cuvettes (Hellma, HL111-10-40) of 1 cm optical path length. The measurements were performed using an Edinburgh nF900 spectrometer, equipped with a CD900 router and a 370 nm diode controlled by IBH NanoLED a driver operating at a 300 kHz repetition rate. The diode was positioned in a standard 90-degree configuration relative to the monochromator entrance and was focused on the cuvette surface. The measurements were performed in the presence of a 420 nm cutoff filter (Thorlabs, FGL420S-2) placed on the emission side. The monochromator was set to collect emission at 520 nm, with slit widths adjusted to 3 nm. Data acquisition was conducted using 1024 channels of the counting card, corresponding to a time window of 0–62.5 ns. The count rate was carefully maintained below 3000 counts per second, and data collection continued until 2000 counts were recorded at the peak. A reference instrument response function (IRF) measurement was performed at the end of each measurement series. This was carried out using an aqueous micelle solution, with the cutoff filter removed, the detection wavelength set to 370 nm, and the monochromator slit widths unchanged. Data acquisition was performed using F900 ver. 5.13 software.

Fluorescence Lifetime Calculation for Solution, Suspensions and Solid States. Lifetime decay and IRF curves were globally normalized within a 0 – 1 scale and deconvoluted by subtracting the IRF signal using a custom Python script based on the Richardson-Lucy deconvolution method. The deconvoluted decay data were then transferred to OriginPro 2024b, where they were fitted using an exponential decay function. The fitting process was constrained to the lowest number of decay components necessary for an adequate fit. In this case, a two-component exponential model provided a satisfactory fit, yielding an $R^2 > 0.9$. The deconvoluted decay data and their respective fits were used to generate the final plots without further modifications.

Upconversion Time-Resolved Emission Measurements. Time-resolved upconversion emission was measured using the previously described pulsed excitation system. Emission centered at 450 nm was spectrally selected by a CM112 monochromator positioned on the detection arm in a standard 90° collection geometry relative to the excitation beam. A small fraction of the excitation light, reflected before the sample, was directed to a fast Si photodiode (DET10A2, Thorlabs) to provide a timing reference. The photodiode trigger and the monochromator output were acquired as separate channels on a Yokogawa DL9140 digital oscilloscope. Signal acquisition and on-scope averaging were performed with the oscilloscope's built-in software, with 1024 waveform integrations per trace. After averaging, data from both channels were exported as ASCII (.txt) files directly from the oscilloscope and transferred to a PC for analysis. Each exported record contained 24 999 uniformly spaced time channels.

Upconversion Time-Resolved Emission Calculation. Oscilloscope traces were imported directly into OriginPro 2024b (OriginLab) without additional preprocessing or smoothing. For each record, the rising segment (from the trigger to the intensity maximum) was isolated and fit using an exponential decay model by nonlinear least squares (default settings) to obtain the rise (negative) amplitude A_1 and time constant τ_1. Next, a global fit to the full time window (rise + decay) was performed with a composite model consisting of the previously determined rise term and two more exponential decays; during this step A_1 and τ_1 were held fixed at the values obtained from the rise-only fit (parameters locked in Origin). Fits were evaluated by inspection of residuals and standard metrics reported by Origin; the lowest-complexity decay model that provided an adequate description was retained. The resulting fitted curves and corresponding raw data were used to generate the final figures without further modification.

Raman Spectroscopy Measurements. Raman spectroscopy measurements were performed *in situ* using Raman microscopy realized by the Renishaw system inVia, a Leica microscope DM2500M, and a Leica objective 50×/0.75 N PLANE EPI. A He-Ne 633 nm laser (Renishaw) was used as the excitation source, with laser power set in the range of 3–5 mW. Measurements were conducted on solid powdered compounds dispersed on a coverslip, recording a Raman map with a minimum area of 20 µm × 20 µm, a 1 µm step size, and an integration time of 1 spectrum/s within the spectral range of 0–1800 cm^{-1}. The measurement area was selected to be representative of the entire sample, ensuring that the mapping was performed within a single granule of the powder. Data acquisition was carried out using the WiRe ver. 5.6 software, installed on a Windows 11 operating system.

Raman Spectroscopy Data Processing. The data, recorded in .wdf format, were processed using WiRe version 5.6. The processing began with loading all spectra corresponding to individual measurement points within the mapped area. Using the trimming function, the peak originating from the laser line was

removed. Next, a component analysis was performed, selecting only the components that contributed significantly across the entire mapped surface. Baseline correction was applied using a 12th-degree polynomial fitting, with the noise level set to 2. The processed data were then smoothed using the Savitzky-Golay method with a 5th-degree polynomial and a window size of 15. Afterward, the spectra from all measurement points were averaged and globally normalized within a 0–1 intensity scale. The final normalized intensity vs. Raman shift data were exported as an ASCII file and imported into OriginPro 2024b. No further processing was applied, and the data were directly used for plotting the final graphs.

Infrared Spectroscopy Measurements. FTIR spectra were collected on a Nicolet iS10 FTIR spectrometer (Thermo Scientific) using a Smart iTX ATR accessory at a resolution of 2 cm^{-1}.

Computational Modeling. Initial models representing *hxl-a*, *p-hcb*, and *m-hcb* topologies were constructed by drawing single-layer structures in ACD/ChemSketch (2021 2.1). The 3D Optimization function was applied to orient aryl-aryl fragments and to achieve the chair conformation of the piperazine units. Subsequently, Mercury software (2022.3.0) was used to measure the distances and angles within the repeating units, and to extract the atomic coordinates belonging to them. The resulting structure, containing a single layer of the unoptimized COF structure, was then used to build a two-layer model: the structure was doubled in the *z* direction with a 6.15 Å interlayer separation. One layer was shifted by vectors ½ *a*, ½ *b*, and ½ *a* + ½ *b*, resulting in one AA stacking mode (no shift) and three AB stacking modes for each COF topology considered. Both cell parameters and atomic coordinates were then optimized with no constraints applied.

All calculations were performed using the CP2K/Quickstep module within the Gaussian and plane wave (GPW) framework.[58,59]

Initial Screening (PBE level). Candidate structures were optimized using the Perdew–Burke–Ernzerhof (PBE) exchange correlation functional[60] combined with Grimme's D3(BJ) dispersion correction.[72] Goedecker–Teter–Hutter (GTH) pseudopotentials were used together with MOLOPT-TZVP basis sets.[62,63] The auxiliary plane-wave expansion employed a cutoff of 400 Ry and a relative cutoff of 40 Ry, with five multigrids. The self-consistent field (SCF) procedure was converged to 10^{-6} Ha, using the orbital transformation (OT) method with a conjugate-gradient minimizer and full preconditioning. Geometry optimizations were performed with the BFGS algorithm until the maximum force on any atom was below 5×10^{-4} Ha·$Bohr^{-1}$.

Final Refinement (PBE0 level). The most stable candidate from the PBE screening was re-optimized at the hybrid level using the PBE0 functional,[64] which mixes 25% Hartree–Fock exchange with 75% PBE exchange and full PBE correlation. The truncated Coulomb operator, as implemented in CP2K, was employed for the treatment of exact exchange. The same basis sets, pseudopotentials, cutoffs, dispersion correction (DFT-D3 with Becke–Johnson damping), and convergence criteria as in the PBE stage were used.

Pores size distribution calculation. Theoretical surface area were calculated from structure data using Zeo++® (version 0.3) with a 1.67 Å probe radius (N_2 molecule), the high-accuracy flag, and 100,000 Monte

Carlo samples per unit cel.[65] DFT-optimized structures of **Ant-COF-H** and **Ant-COF-OH** representing AA and AB stacking modes of *p-hcb* topology have been used as input files.

Kinetic model. The excitation–power dependence of TTA upconversion is governed by the competition between **first-order** triplet loss (unimolecular decay/quenching) and **second-order** triplet–triplet annihilation (TTA). At low excitation power densities, annihilator triplets are sufficiently dilute that their dominant fate is first-order decay, leading to an approximately quadratic dependence of upconverted emission on power ($I_{\mathrm{UC}} \propto P^2$). At higher excitation densities, the bimolecular TTA pathway becomes competitive or dominant, and the power dependence approaches linearity ($I_{\mathrm{UC}} \propto P$).

To rationalize the excitation–power dependence of the upconverted emission, we implemented a steady-state kinetic model for a sensitized triplet–triplet annihilation upconversion (TTA-UC) system under continuous-wave (CW) excitation. The model couples (i) photon absorption by the sensitizer, (ii) sensitizer triplet generation by intersystem crossing (ISC), (iii) diffusion-influenced triplet–triplet energy transfer (TTET) from sensitizer to annihilator, and (iv) annihilator triplet fate via first-order decay and bimolecular TTA, followed by singlet formation and fluorescence. For each excitation power density $P(\mathrm{mW\ cm^{-2}})$, the model predicts the steady-state sensitizer triplet concentration $[T_S]$, annihilator triplet concentration $[T_A]$, and the corresponding upconversion emission intensity $I_{\mathrm{UC}}(P)$. The apparent power-law exponent $n(P)$ is then obtained from the slope of the simulated $\log I_{\mathrm{UC}}$ versus $\log P$ curve.

Absorption and sensitizer triplet generation

The absorbed fraction of the excitation beam is represented either by Beer–Lambert attenuation,

$$f_{\mathrm{abs}} = 1 - 10^{-\varepsilon\, c_S\, \ell} \qquad \text{(eq. S6)}$$

or by a user-defined absorption efficiency (used when optical scattering or effective absorption is treated phenomenologically). Here ε is the sensitizer molar absorption coefficient ($\mathrm{M}^{-1} cm^{-1}$), c_S the sensitizer concentration (M), and ℓ the optical path length (cm). For a given power density P, the absorbed photon rate is converted to a volumetric absorption rate $R_{\mathrm{abs}}(P)$ in units of $\mathrm{M}\ s^{-1}$ as,

$$R_{\mathrm{abs}}(P) = \frac{f_{\mathrm{abs}} \cdot P \cdot \lambda}{\ell\ \cdot hc \cdot \mathrm{N_A}} \qquad \text{(eq. S7)}$$

using photon energy and the sample geometry. Sensitizer triplets are generated with yield ϕ_{ISC},

$$G_{T_S}(P) = \phi_{\mathrm{ISC}}\, R_{\mathrm{abs}}(P) \qquad \text{(eq. S8)}$$

where $G_{T_S}$is the sensitizer triplet generation rate ($\mathrm{M}\ cm^{-1}$).

Diffusion-influenced TTET and TTA rate constants

Diffusion coefficients are either provided directly or computed from the Stokes–Einstein relation,

$$D = \frac{k_B T}{6\pi\eta r} \qquad \text{(eq. S9)}$$

where T is temperature, η is viscosity, and r is a hydrodynamic radius. TTET and TTA are allowed to use distinct radii (and thus diffusion coefficients), reflecting different effective sizes of the diffusing partners.

For an encounter-controlled bimolecular process with capture radius R, the Smoluchowski diffusion-limited rate constant is

$$k_{\mathrm{diff}} = 4\pi R\, D_{\mathrm{rel}}\, N_A\, 10^3 \quad \text{(eq. S10)}$$

where $D_{\mathrm{rel}} = D_1 + D_2$ is the relative diffusion coefficient ($m^2\ s^{-1}$), $N_A$is Avogadro's constant, and the factor 10^3converts m^3 to L, yielding k_{diff}in $\mathrm{M}^{-1}s^{-1}$. Finite intrinsic reactivity is incorporated with Collins–Kimball kinetics:

$$k_{\mathrm{eff}} = \left(\frac{1}{k_{\mathrm{diff}}} + \frac{1}{k_{\mathrm{intr}}}\right)^{-1} \quad \text{(eq. S11)}$$

which is applied separately to obtain an effective TTET constant k_{TTET} and an effective TTA constant k_{TTA}.

Steady-state triplet balances with self-consistent ground-state availability

Triplet lifetimes enter explicitly through first-order decay constants. We define effective first-order loss rates (including optional additional quenching/thermal dissipation terms k_q):

$$k_{T_S} = \tau_{T_S}^{-1} + k_{q,S}, k_{T_A} = \tau_{T_A}^{-1} + k_{q,A} \quad \text{(eq. S12)}$$

where $\tau_{T_S}$and $\tau_{T_A}$are the sensitizer and annihilator triplet lifetimes, respectively.

Under CW excitation, the sensitizer triplet concentration is obtained from the steady-state balance between generation, first-order decay, and TTET quenching by *ground-state* annihilators:

$$0 = G_{T_S}(P) - k_{T_S}[T_S] - k_{\mathrm{TTET}}[T_S][A_{\mathrm{free}}] \quad \text{(eq. S13)}$$

giving

$$[T_S](P) = \frac{G_{T_S}(P)}{k_{T_S} + k_{\mathrm{TTET}}[A_{\mathrm{free}}]} \quad \text{(eq. S14)}$$

Annihilator triplets are generated by TTET at rate

$$G_{T_A}(P) = k_{\mathrm{TTET}}[T_S][A_{\mathrm{free}}] \quad \text{(eq. S15)}$$

and are depleted by first-order decay and by bimolecular TTA:

$$0 = G_{T_A}(P) - k_{T_A}[T_A] - 2k_{\mathrm{TTA}}[T_A]^2 \quad \text{(eq. S16)}$$

Equation S16 is solved analytically (quadratic form) to obtain $[T_A](P)$.

A key feature of the model is that TTET requires *ground-state* annihilators; therefore the available annihilator concentration is treated self-consistently as

$$[A_{\text{free}}] = c_A - [T_A] \quad \text{(eq. S17)}$$

where $c_A$is the total annihilator concentration. Equations (S13)–(S16) form a coupled steady-state system because $[A_{\text{free}}]$depends on $[T_A]$, while $[T_A]$depends on the TTET generation rate, which depends on $[A_{\text{free}}]$. We solve this coupling by fixed-point iteration with (i) **continuation** (the converged $[T_A]$ at P_i is initialized from the solution at P_{i-1}), (ii) **under-relaxation** (damped updates) to suppress nonphysical branch switching, and (iii) physical bounds $0 \leq [T_A] \leq c_A$and $0 \leq [T_S] \leq c_S$. These numerical safeguards ensure a smooth transition between the low-power and high-power regimes without artificial collapse of $[T_A]$.

<u>*Upconversion emission intensity*</u>

The TTA event rate (in $\text{M}\ s^{-1}$) is

$$R_{\text{TTA}}(P) = k_{\text{TTA}}[T_A]^2 \quad \text{(eq. S18)}$$

Only a fraction of TTA events yields an emissive singlet. We represent this by a composite singlet yield per TTA event,

$$\eta_{\text{TTA}\to S_1} = \eta_{\text{ET}}\ \eta_{\text{TTA}}\ f_{\text{singlet}} \quad \text{(eq. S19)}$$

where η_{ET} and η_{TTA} are user-specified efficiency factors (0–1) accounting for non-spin losses in the annihilation channel, and f_{singlet} is the spin-statistical singlet fraction. The upconverted photon production rate ($\text{M}\ s^{-1}$) is then

$$R_{\text{UC}}(P) = \phi_F\ \eta_{\text{TTA}\to S_1}\ R_{\text{TTA}}(P) = \phi_F\ \eta_{\text{TTA}\to S_1}\ k_{\text{TTA}}[T_A]^2 \quad \text{(eq. S20)}$$

where ϕ_F is the annihilator fluorescence quantum yield from S_1. Finally, $R_{\text{UC}}(P)$ is converted to an emitted photon flux $I_{\text{UC}}(P)$ in photons $cm^{-2}\ s^{-1}$ using the sample path length ℓ.

<u>*Extraction of the power-law exponent $n(P)$*</u>

To quantify the apparent power dependence, we evaluate the **local** exponent

$$n(P) = \frac{d\log\ I_{\text{UC}}(P)}{d\log\ P} \quad \text{(eq. S21)}$$

In practice, $n(P)$ is computed numerically from the discrete simulated arrays $\{P_i\}$and $\{I_{\text{UC},i}\}$ by taking a finite-difference gradient of $\log I_{\text{UC}}$with respect to $\log P$. In the conventional TTA-UC framework, n approaches ~ 2 when annihilator triplet loss is dominated by first-order decay (low power) and approaches ~ 1 when TTA dominates the triplet decay balance (high power), with intermediate values when both loss channels contribute comparably.

2. Synthesis

9,10-dibromoantharecene (S1)

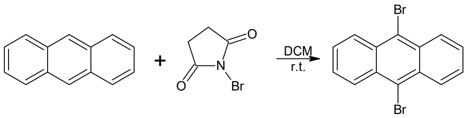

Anthracene (5.0 g, 0.028 mol, 1.0 equiv.) was dissolved in 250 mL of dichloromethane (DCM) at room temperature, and N-bromo succinimide (NBS, 10.24 g, 0.058 mol, 2.05 equiv.) was added in one portion. The reaction mixture was stirred at room temperature for 3 hours, after which the DCM was evaporated. The resulting solid was suspended in methanol (MeOH, 180 mL) and the precipitate was filtered, washed with an additional portion of MeOH (20 mL), and dried to afford a light green solid of 9,10-dibromoanthracene **S1** (8.5 g, 0.0253 mol, 90%). 1H NMR (500 MHz, CDCl3) δ 8.60 (dd, J = 6.8, 3.2 Hz, 1H), 7.65 (dd, J = 6.8, 3.1 Hz, 1H). The characterization data agree with the literature .[66]

2-hydroxy-4-(4,4,5,5-tetramethyl-1,3,2-dioxaborolan-2-yl)benzaldehyde (S2)

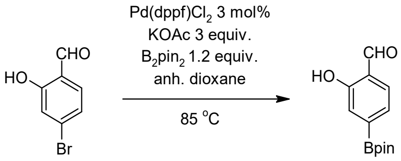

4-bromosalicylaldehyde (6.0 g, 29.85 mmol, 1 equiv.), bis(pinacolato)diboron (9.1 g, 35.82 mmol, 1.2 equiv.), [1,1′-Bis(diphenylphosphino)ferrocene]palladium(II) dichloride (0.66 g, 0.90 mmol, 0.03 equiv.) and potassium acetate (8.8 g, 89.54 mmol, 3 equiv.) were placed into the Schlenk flask, evacuated and refilled with nitrogen three times. Next 75 mL of anhydrous dioxane was added, reaction mixture was additionally degassed and stirred at 85 °C for 16 h. After cooling to room temperature, the solvent was evaporated to dryness under reduced pressure. The residue was dissolved in n-hexane:ethyl acetate mixture (9:1, v/v), the solution was washed with H2O and passed through short silica gel pad which was additionally eluted with n-hexane:ethyl acetate mixture (8:2, v/v). After evaporation the raw material was further purified by recrystallization from hexane to give desired compound **S2** as a colorless solid (6.0 g, 24.18 mmol, 81%). ^{1}H NMR (500 MHz, $CDCl_3$) δ 10.83 (s, 1H), 9.93 (d, *J* = 0.5 Hz, 1H), 7.56 (d, *J* = 0.6 Hz, 1H), 7.42 (m, 2H), 1.35 (s, 12H). The characterization data agree with the literature.[67]

9,10-bis(4-formylphenyl)anthracene (Ant-CHO)

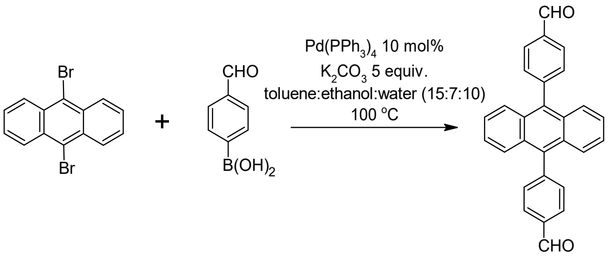

9,10-Dibromoantharecene **S1** (1.016 g, 3.23 mmol, 1 equiv.), *p*-formylphenylboronic acid (1.87 g, 12.50 mmol, 2.1 equiv.), K_2CO_3 (4.11 g, 29.80 mmol, 5 equiv.) and $Pd(PPh_3)_4$ (690 mg, 0.595 mmol, 0.1 equiv.)

were placed into a Schlenk flask containing a mixture of solvents toluene:ethanol:water (30:15:20, v/v/v, 65 mL), degassed, and stirred at 100 °C for 16 hours. After cooling to room temperature, 20 mL of MeOH was added the yellow solid was filtered, washed with water (4x10 mL), MeOH (2x10 mL). Solid was redissolved in DCM and passed through shot silica gel pad. After evaporation product was crystalized from dioxane:toluene mixture (3:2, v/v, 150 mL, reflux). Yellow crystals were filtered, washed with MeOH (2x10 mL) and dried to give desired compound **Ant-CHO** (1.88 g, 4.86 mmol, 82%). ^{1}H NMR (300 MHz, DMSO-d_6) δ 10.25 (s, 2H), 8.23 (d, *J* = 8.0 Hz, 4H), 7.76 (d, *J* = 7.9 Hz, 4H), 7.53 (ddd, *J* = 25.6, 7.1, 3.3 Hz, 8H). The characterization data agree with the literature.[68]

9,10-bis(4-formyl-3-hydroxyphenyl)anthracene (Ant-CHO(OH))

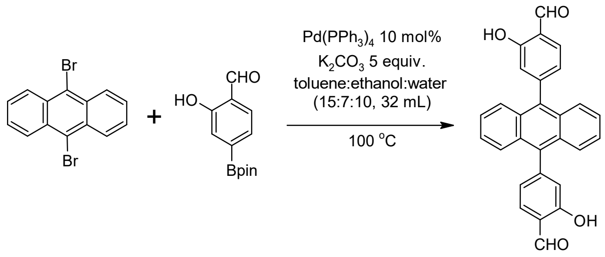

9,10-Dibromoanthracene **S1** (2.0 g, 5.95 mmol, 1 equiv.), 2-hydroxy-4-(4,4,5,5-tetramethyl-1,3,2-dioxaborolan-2-yl)benzaldehyde **S2** (1.65 g, 6.65 mmol, 2.2 equiv.), K_2CO3 (2.09 g, 15.12 mmol, 5 equiv.), and Pd(PPh3)4 (349 mg, 0.59 mmol, 0.1 equiv.) were added to a Schlenk flask containing a mixture of toluene (14 mL), ethanol (7 mL), and water (10 mL). The reaction mixture was degassed and stirred at 110 °C for 16 hours. After completion, the reaction was poured into DCM (50 mL) and washed with 6% aq. HCl (20 mL). The mixture was filtered through a short silica gel pad using DCM as the eluent. The solvent was concentrated, and the resulting solid was washed with a DCM/hexane mixture (1:2, v/v, 25 mL). The crude product was collected by filtration and air-dried, yielding 1.17 g (84%). Initial attempts to use this product for aminal-COF synthesis via solvothermal methods resulted in black solid materials. Therefore, further purification was performed by column chromatography using a gradient elution of DCM/hexane (from 1:1 to 2:1, v/v, silica gel). Finally, product **Ant-CHO(OH)** was isolated as pale yellow solid (1.04 g, 2.48 mmol, 75%). ^{1}H NMR (600 MHz, $CDCl_3$) δ 11.24 (s, 1H, *syn*), 11.24 (s, 1H, *anti*), 10.07 (s, 1H), 7.79 (dd, J = 7.9, 3.9 Hz, 1H), 7.68–7.64 (m, 2H), 7.37 (dd, J = 6.9, 3.2 Hz, 2H), 7.17 – 7.12 (m, 2H). The characterization data agree with the literature.[69]

Ant-COF-H. Aldehyde Ant-CHO (96.61 mg, 0.25 mmol, 1 equiv.) and piperazine (43.07 mg, 0.5 mmol, 2 equiv.) were placed into 8 mL screw-cap vials, transferred to glovebox (nitrogen atmosphere), and combined with 4 mL of anhydrous dioxane. The vial was then screw and transferred out of the glovebox. The reaction mixture was then heated in pre-warmed aluminum blocks at 120 °C for 3 days. After completion, the solid product was filtered and washed sequentially with acetone, DCM, and hexane, then dried under dynamic vacuum for at least 4 hours. Product was obtained as a light white powder (131 mg, 93% yield). Elemental analysis calcd for $C_{72}N_8H_{68}$: C, 82.67; N, 10.73; H, 6.51. Found C, 79.77; N, 9.41; H, 7.63.

Ant-COF-OH. Aldehyde Ant-CHO(OH) (104.61 mg, 0.25 mmol, 1 equiv.) and piperazine (43.07 mg, 0.5 mmol, 2 equiv.) were placed into 8 mL screw-cap vials, transferred to glovebox (nitrogen atmosphere), and combined with 4 mL of anhydrous dioxane. The vial was then screw and transferred out of the glovebox. The reaction mixture was then heated in pre-warmed aluminum blocks at 120 °C for 3 days. After completion, the solid product was filtered and washed sequentially with acetone, DCM, and hexane, then dried under dynamic vacuum for at least 4 hours. Product was obtained as a light yellowish powder (124 mg, 89% yield). Elemental analysis calcd for $C_{72}N_8O_4H_{68}$: C, 77.98; N, 10.11; H, 6.14. Found C, 76.11; N, 9.39; H, 7.37.

3. COF synthesis optimization

For the synthesis of **Ant-COF-H** and **Ant-COF-OH**, the corresponding aldehyde (**Ant-CHO** or **Ant-CHO(OH)**, 0.25 mmol, 1 equiv.) and piperazine (43.07 mg, 0.5 mmol, 2 equiv.) were placed into 8 mL screw-cap vials, transferred to glovebox (nitrogen atmosphere), and combined with 4 mL of anhydrous solvent. The reaction mixtures were then heated in pre-warmed aluminum blocks at 120 °C for 3 days. After completion, the solid products were filtered and washed sequentially with acetone, DCM, and hexane, then dried under dynamic vacuum for at least 4 hours.

The PXRD patterns for these materials obtained in various solvents, along with isolated yields, are shown in Figure S1. For **Ant-COF-H**, PXRD analysis confirmed good crystallinity in samples synthesized with *o*-dichlorobenzene (ODCB), n-BuOH, and dioxane (highest yield). Similarly, the synthesis of **Ant-COF-OH** in both dioxane and ODCB resulted in materials with comparable crystallinity and yields. Dioxane was chosen as the optimal solvent, ensuring the integrity of the synthesis conditions for both COFs and minimizing the risk of material contamination with a low-volatile solvent such as ODCB.

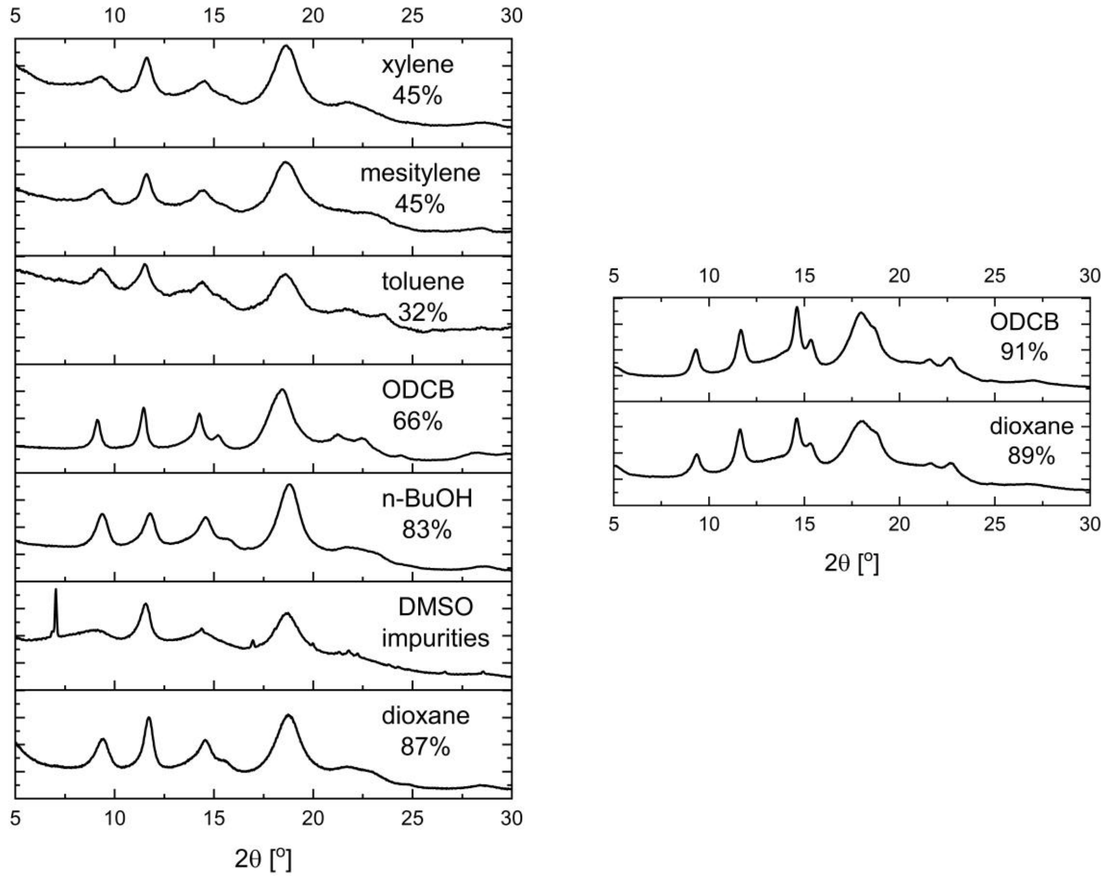

Fig S1. PXRD spectra and isolated yields of **Ant-COF-H** (left) and **Ant-COF-OH** (right) from solvent screening studies.

4. PXRD

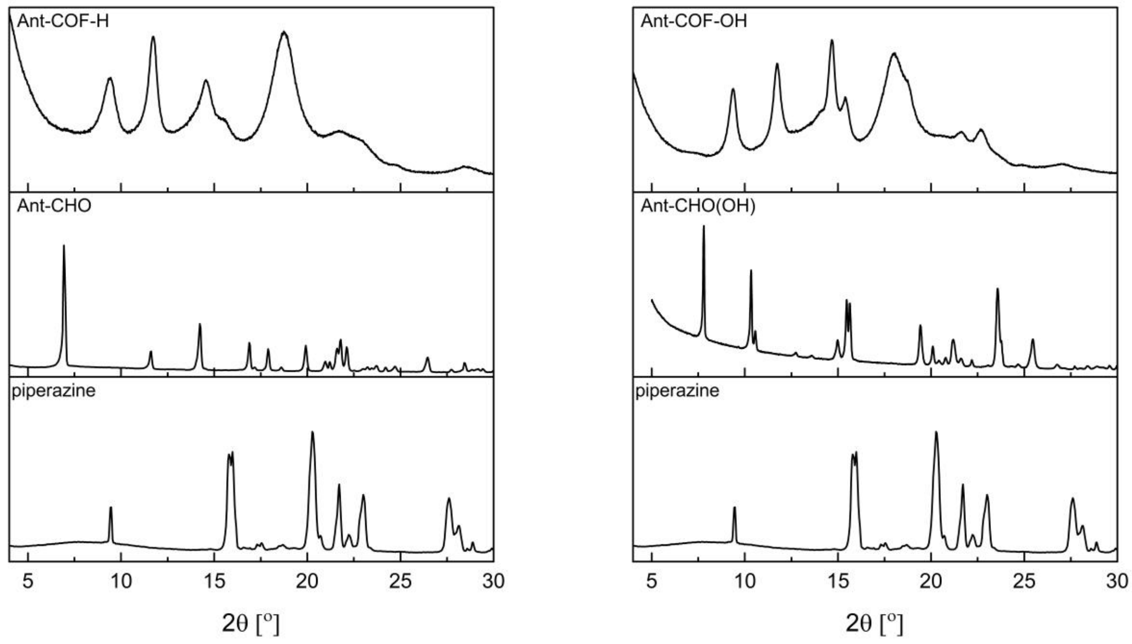

Fig S2. Comparison of PXRD patterns of the **Ant-COF-H** (left) and **Ant-COF-OH** (right) with corresponding substates used for its synthesis: **Ant-CHO**, **Ant-CHO(OH)**, and piperazine.

5. Structure elucidation

Table S1. Initial structure optimization convergence (PBE) for **Ant-COF-H** and **Ant-COF-OH** topologies.

stacking mode	Topologies convergence		
	p-hcb	***hxl-a***	***m-hcb***
**AA**	yes	no	no
**AB**	yes	no	no

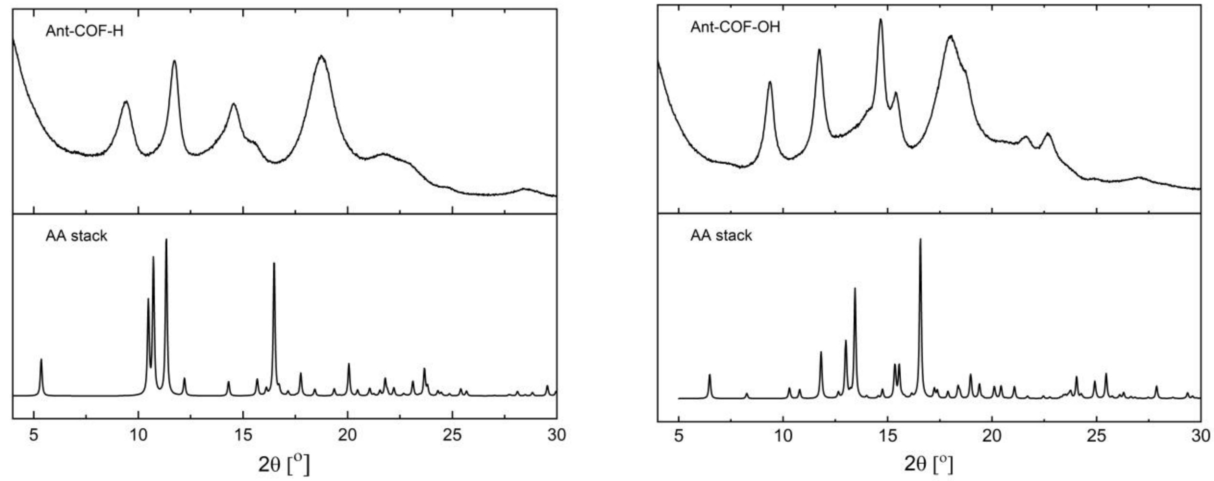

Figure S3. Comparison between experimental and simulated PXRD patterns for **AA** stack models (PBE) in *p-hcb* topology of **Ant-COF-H** (left) and **Ant-COF-OH** (right).

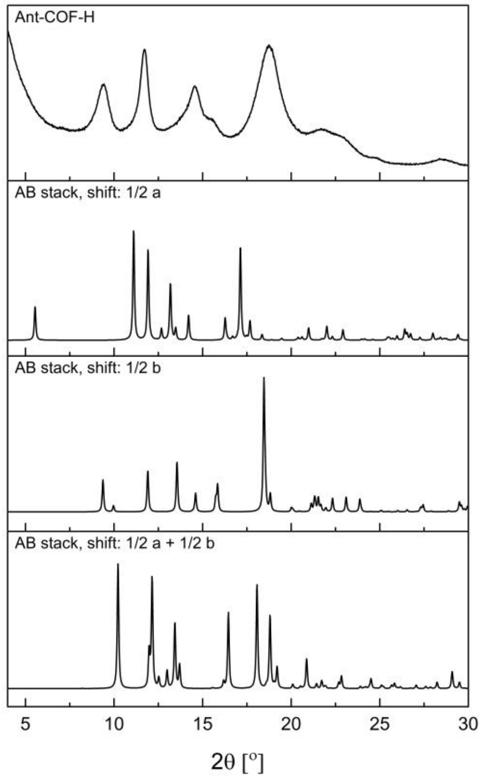

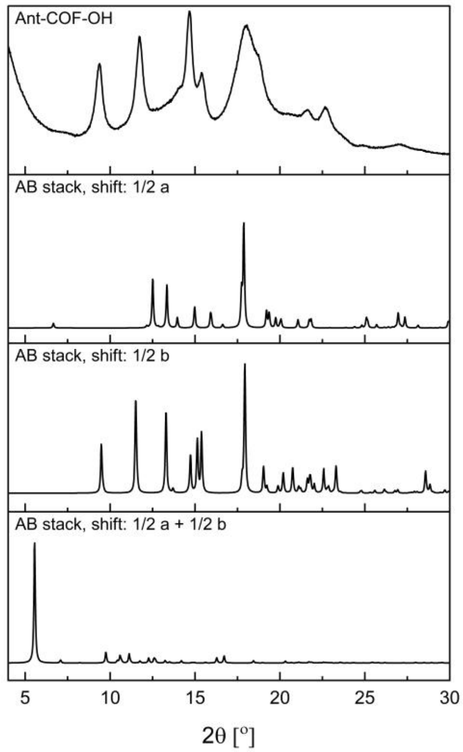

Figure S4. Comparison between experimental and simulated PXRD patterns for **AB** stack models of **Ant-COF-H** (left) and **Ant-COF-OH** (right) in *p-hcb* topology (PBE). COF's layer shift in *a*, *b*, and both directions were considered.

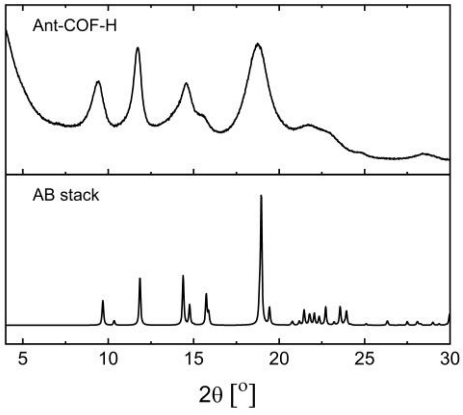

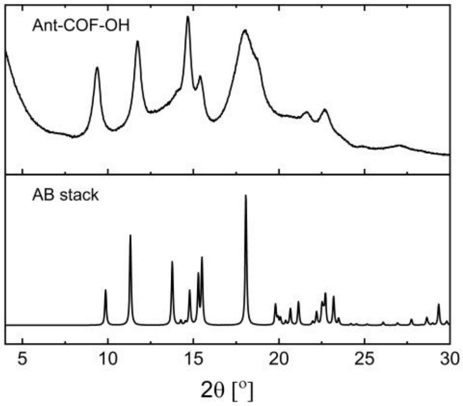

Figure S5. Comparison between experimental and simulated PXRD patterns for AB stack models in *p-hcb* topology obtained at the PBE(0) level calculations for **Ant-COF-H** (left) and **Ant-COF-OH** (right). In both cases optimized structures of AB stack with 1/2b layer shifts were used as a starting geometry for calculations.

Table S2. Calculated and refined lattice parameters for **Ant-COF-H** and **Ant-COF-OH** for AB stack and ½ b layer shift.

lattice parameters	**Ant-COF-H**		**Ant-COF-OH**	
	calculated model	refined parameters	calculated model	refined parameters
a [Å]	9.152	9.980	8.98	9.608
b [Å]	15.144	16.553	15.788	16.785
c [Å]	9.472	10.355	9.929	10.616
α [°]	98.83	113.89	98.07	116.19
β [°]	90.27	79.22	93.21	80.53
γ [°]	94.12	91.67	91.45	91.60
reliability factors	R_p = 5.02<br>R_{wp} = 4.70<br>R_{exp} = 3.82<br>χ^2 = 1.51	Rp=4.45<br>Rwp=4.27<br>R_{exp} = 3.12<br>χ^2 = 1.87		

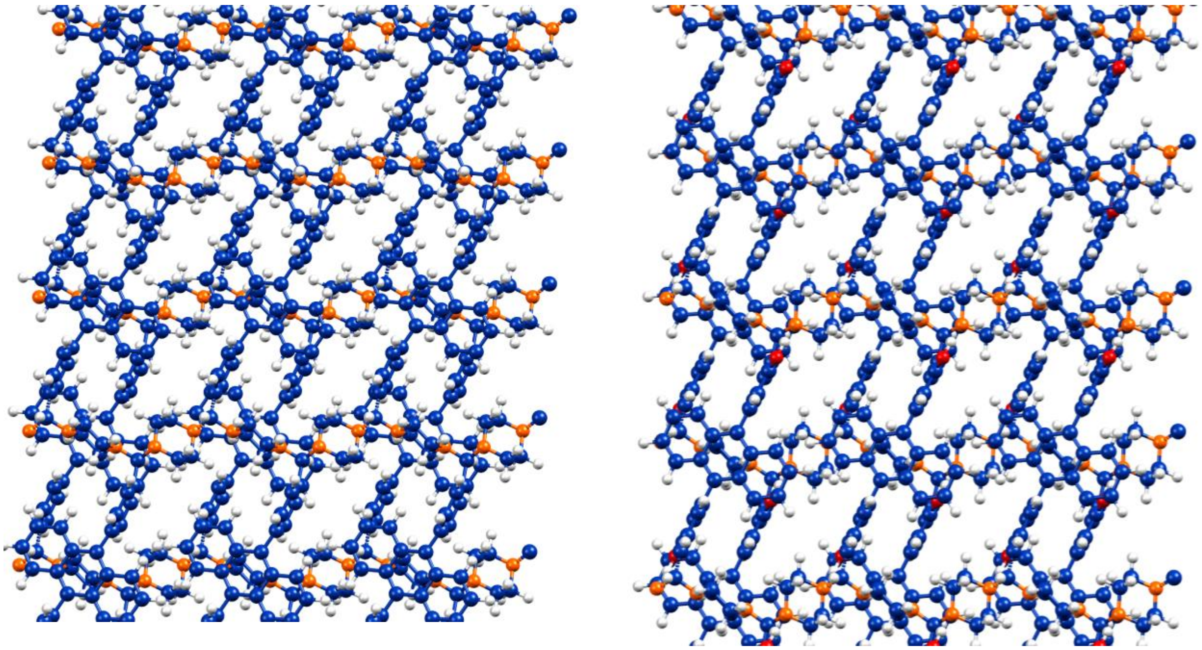

Figure S6. Calculated structural packings of **Ant-COF-H** and **Ant-COF-OH**, view along c-axis; color coding of atoms: blue – carbon, orange – nitrogen, red – oxygen, white – hydrogen.

Table S3. Calculated structural parameters (Zeo++) for **Ant-COF-H** and **Ant-COF-OH** considering their most stable AA and AB stacking modes. ASA: accessible surface area per units cell, NASA: non-accessible surface area per unit cell.

	**Ant-COF-H stack AA**	**Ant-COF-H stack AB**	**Ant-COF-OH stack AA**	**Ant-COF-OH stack AB**
Unit cell volume [Å^3]	1666.52	1293.72	1505.22	1390.79
Density [g/cm^3]	1.04	1.34	1.22	1.32
ASA [Å^2]	0	0	0	0
NASA [Å^2]	27.9273	0	0	0
Number of channels	0	0	0	0
Number of pockets	4	0	0	0
Pocket surface area [Å^2]	7.08, 6.98, 6.95, 6.91	0	0	0

6. Infrared spectra

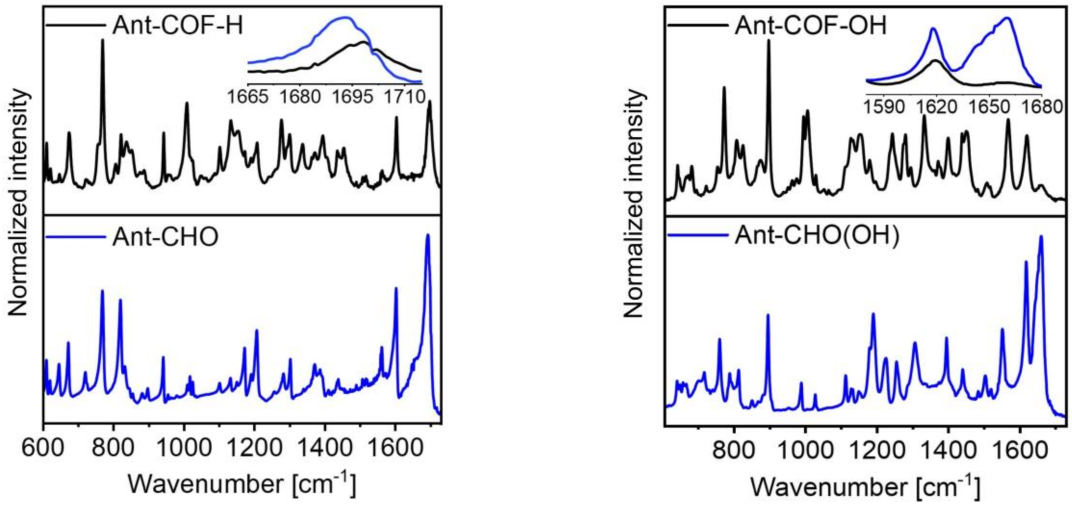

Figure S7. Infrared spectra of COFs and used substates: **Ant-COF** (left) and **Ant-COF-OH** (right).

7. TEM

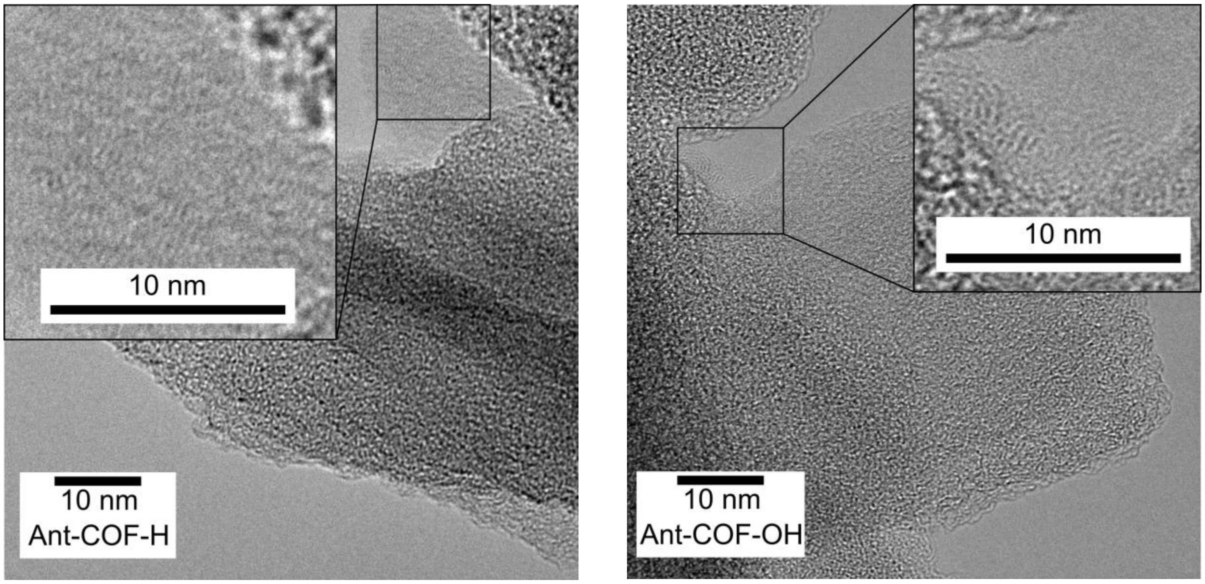

Figure S8. High magnification TEM images of **Ant-COF-H** (left) and **Ant-COF-OH** (right). Insets: regions revealing periodic contrast modulation.

8. DSC-TGA

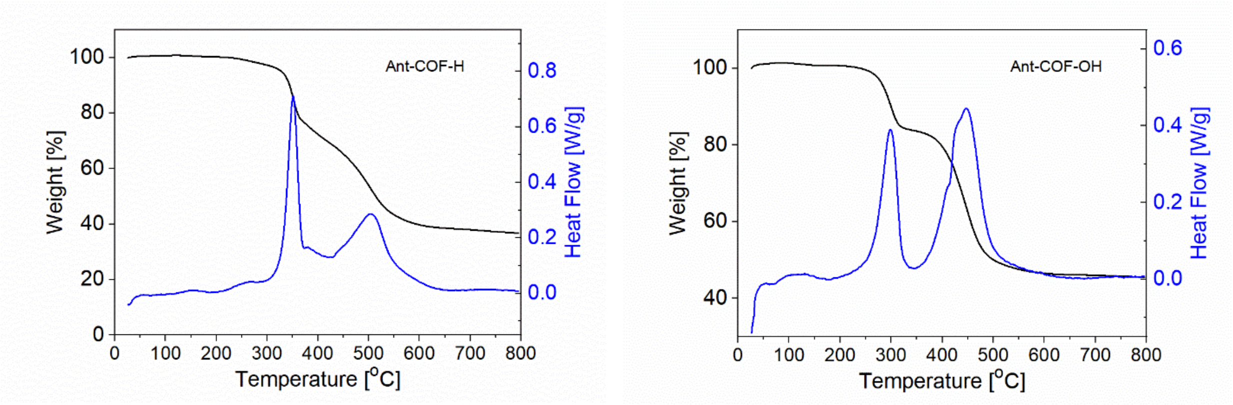

Figure S9. DSC-TGA graph representing thermal stability of **Ant-COF-H** (left) and **Ant-COF-OH** (right).

9. Spectroscopy

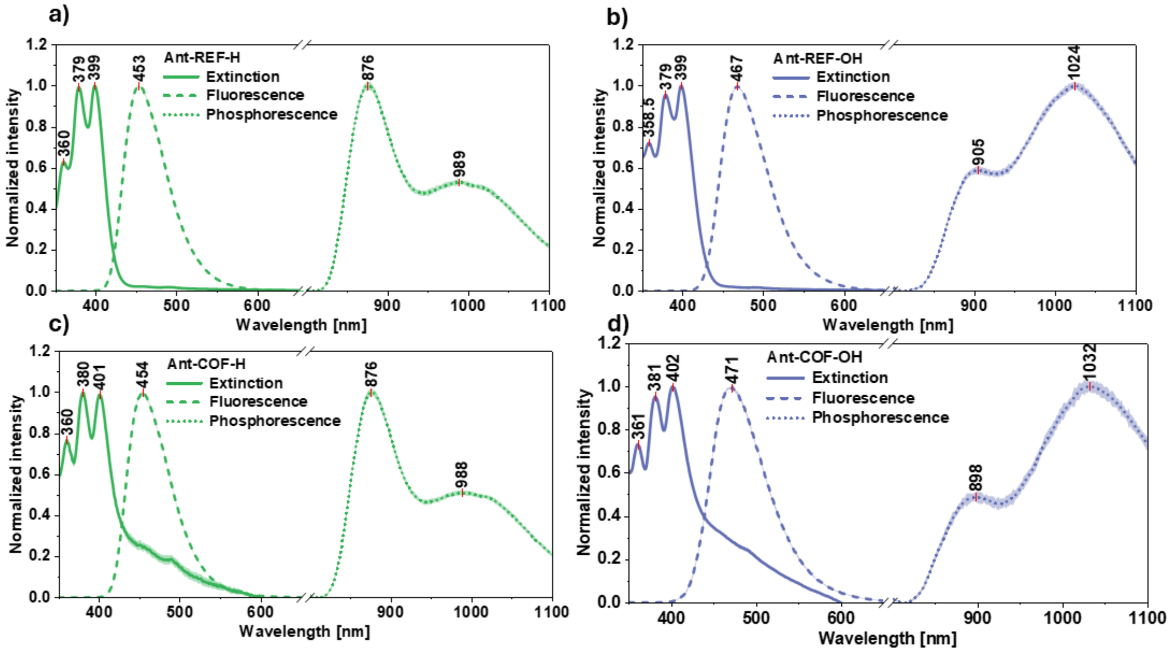

Figure S10. Comparison of extinction, fluorescence, and phosphorescence spectra of substrates: **Ant-CHO** (a) **Ant-CHO(OH)** (b), and COFs: **Ant-COF-H** (c) **Ant-COF-OH** (d) in 1,2-dibromoethane.

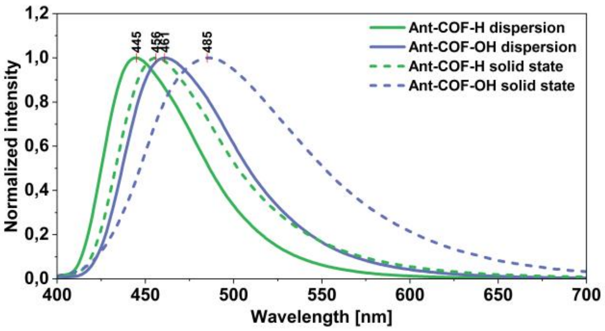

Figure S11. Comparison of fluorescence spectra of COFs **Ant-COF-H** and **Ant-COF-OH** in solid state and in toluene dispersion.

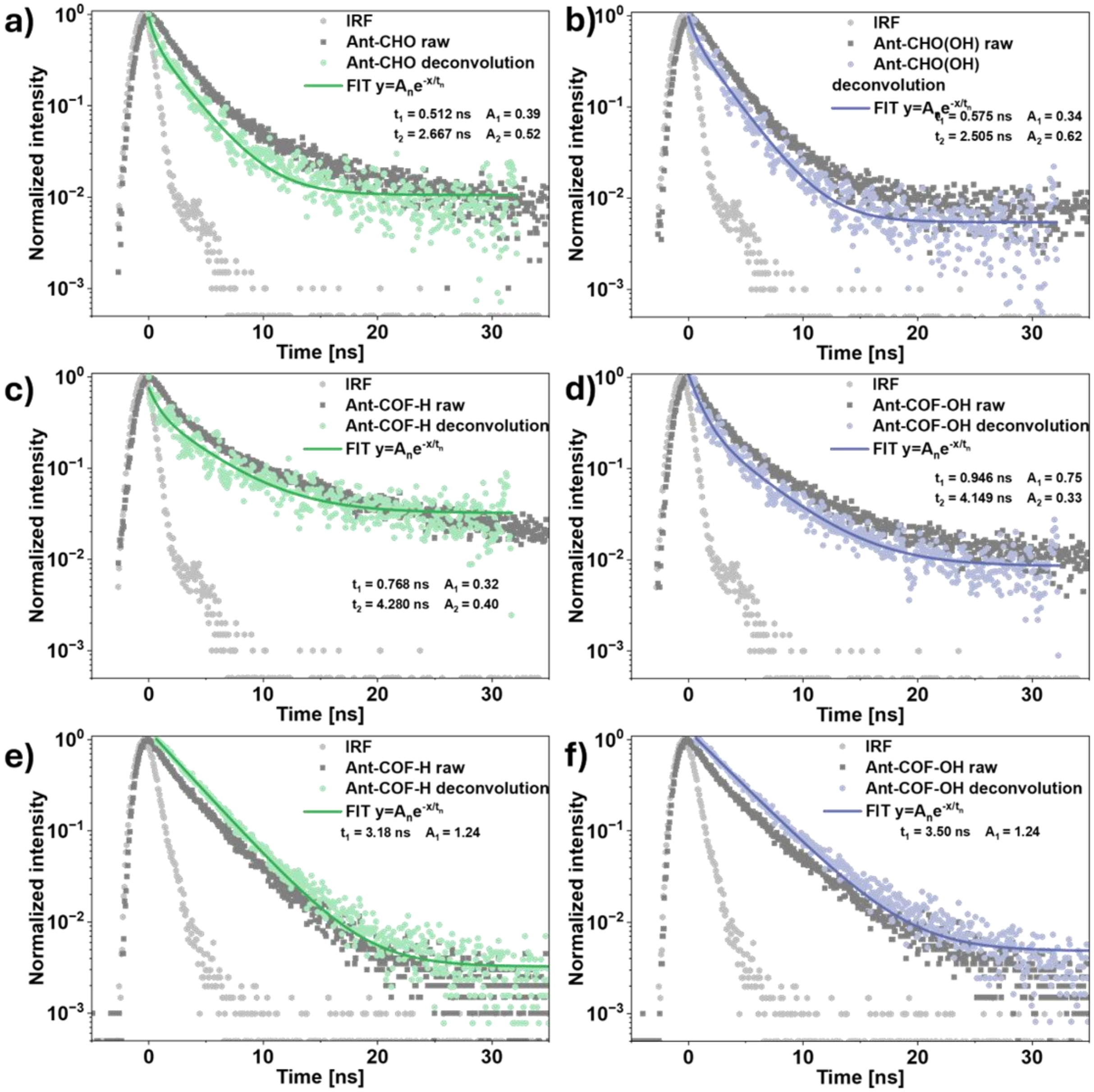

Figure S12. Time-resolved fluorescence spectra: of substrates solutions in toluene: **Ant-CHO** (a), **Ant-CHO(OH)** (b); COFs in solid state: **Ant-COF-H** (c) **Ant-COF-OH** (d); and COFs dispersion in toluene: **Ant-COF-H** (e) and **Ant-COF-OH** (f).

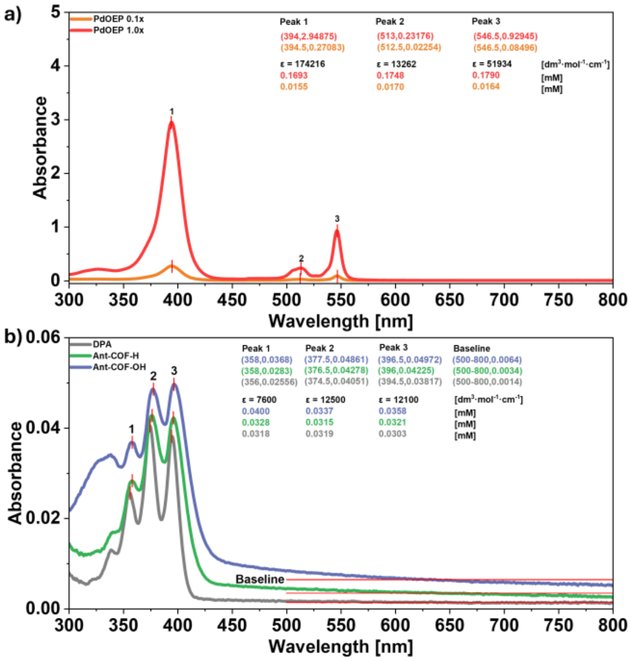

Figure S13.(a) Absorption spectra of **PdOEP** toluene solution in two concentration regimes; (b) COFs **Ant-COF-H** and **Ant-COF-OH** suspensions in toluene with comparison to **DPA** toluene solution.

10. Concentration screening for the upconversion

Initially, the **Ant-COF-H** upconversion system with varying sensitizer and annihilator concentrations was investigated. In total, 4 combinations were tested (Table S4) – two concentrations of PdOEP ($0.1\mathrm{x}$ – $0.017mM$ and $1\mathrm{x}$ – $0.17mM$) and two concentrations of COF ($0.1\mathrm{x}$ – $0.032mM$ and $1\mathrm{x}$ – $0.32mM$), with the maximal COF concentration $1\mathrm{x}$ (expressed as the average diphenylanthracene unit concentration, calculated from the absorption coefficient of diphenylanthracene, Figures S13b), yielding a stable suspension (Figure S14). Optimal performance yielding the highest upconversion quantum yield at threshold power density and a relatively low power law power exponent of emission intensity dependent on power density was observed at $1x$ **Ant-COF-H**and 0.1x **PdOEP** concentrations. For further discussion, the **Ant-COF-H–PdOEP** and **Ant-COF-OH–PdOEP** systems were selected at matched concentrations of $0.1\mathrm{x}$ **PdOEP** and $0.1\mathrm{x}$ COFs. This selection was based on the observation that the **Ant-COF-H** system performed comparably well at this lower concentration, while the maximal usable concentration of **Ant-COF-OH** was limited by suspension stability, precluding the use of a $1\mathrm{x}$ concentration.

Table S4. Annotations used for different **Ant-COF-H** – **PdOEP** solutions compositions.

	**PdOEP**, $0.17\ mM$	**PdOEP**, $0.017\ mM$
**Ant-COF-H**, $0.32\ mM$	1xAnt-COF-H 1xPdOEP	1xAnt-COF-H 0.1xPdOEP
**Ant-COF-H**, $0.032\ mM$	0.1xAnt-COF-H 1xPdOEP	0.1xAnt-COF-H 0.1xPdOEP
**Ant-COF-OH**, $0.037\ mM$	-	0.1xAnt-COF-H 0.1xPdOEP
**DPA,** $0.032\ mM$	-	DPA 0.1 PdOEP

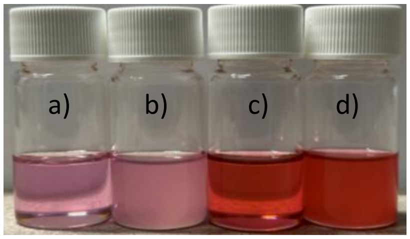

Figure S14. Suspensions of COF **Ant-COF-H** with **PdOEP** in various proportions and concentartion regimes: (a) 0.1x**Ant-COF-H** 0.1x**PdOEP**, (b) 1x**Ant-COF-H** 0.1x**PdOEP**, (c) 0.1x**Ant-COF-H** 1x**PdOEP**, (d) 1x**Ant-COF-H** 1x**PdOEP**.

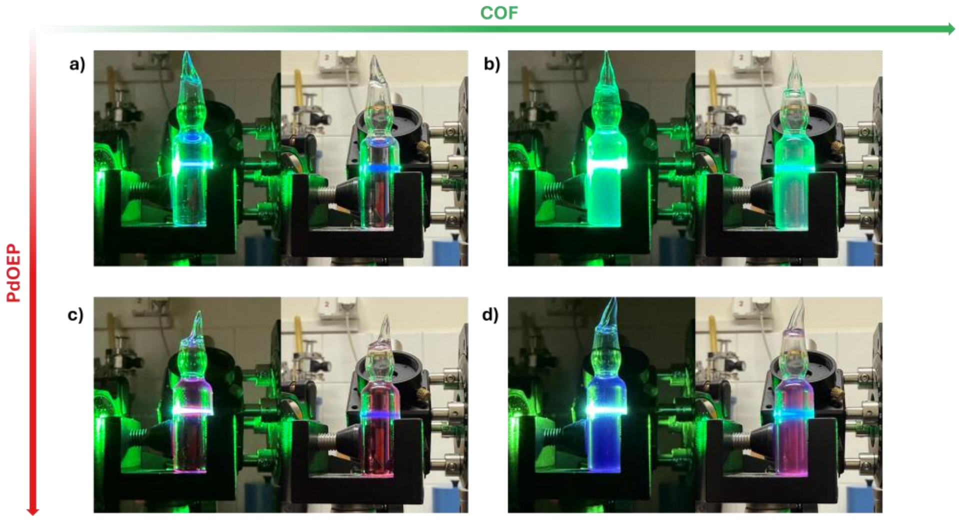

Figure S15. Blue TTA upconverted emission in flame-sealed ampoules containing samples of varying composition and concentration of COF **Ant-COF-H** and **PdOEP** under green laser excitation, shown with light on and off: (a) 0.1x**Ant-COF-H** 0.1x**PdOEP**, (b) 1x**Ant-COF-H** 0.1x**PdOEP**, (c) 0.1x**Ant-COF-H** 1x**PdOEP**, (d) 1x**Ant-COF-H** 1x**PdOEP**.

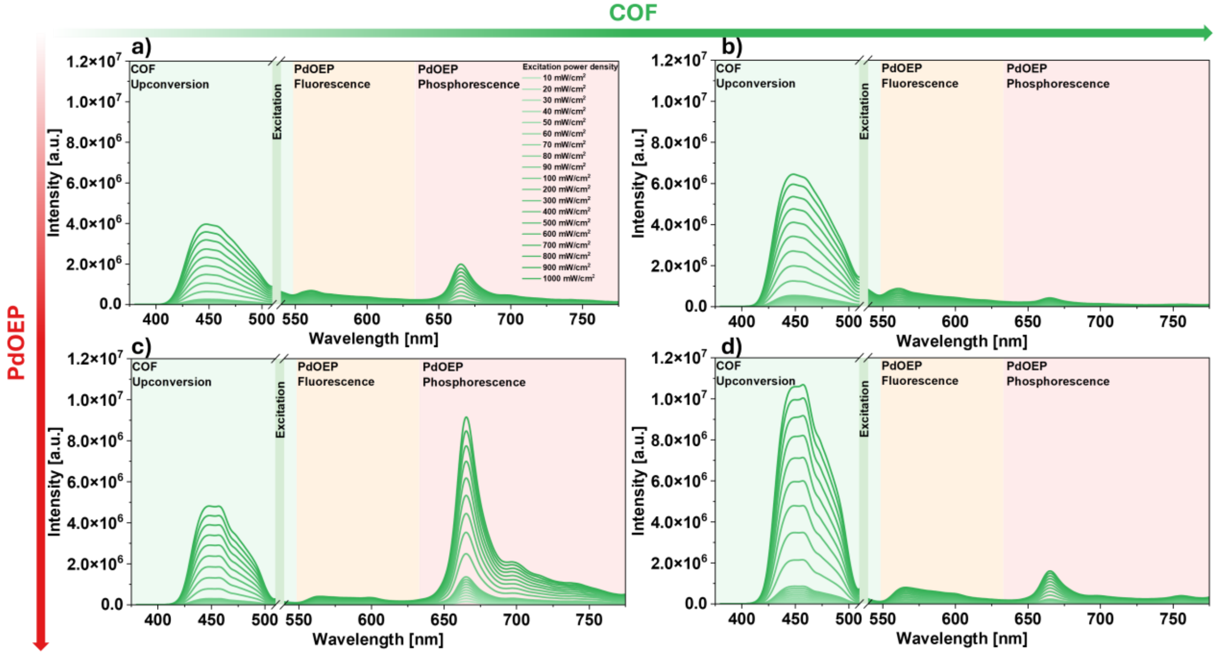

Figure S16. Photoluminescence spectra of samples with varying composition and concentration of COF **Ant-COF-H** and **PdOEP** under excitation at 523 nm with power densities 10–1000 mW·cm^{-2}: (a) 0.1x**Ant-COF-H** 0.1x**PdOEP**, (b) 1x**Ant-COF-H** 0.1x**PdOEP**, (c) 0.1x**Ant-COF-H** 1x**PdOEP**, (d) 1x**Ant-COF-H** 1x**PdOEP**.

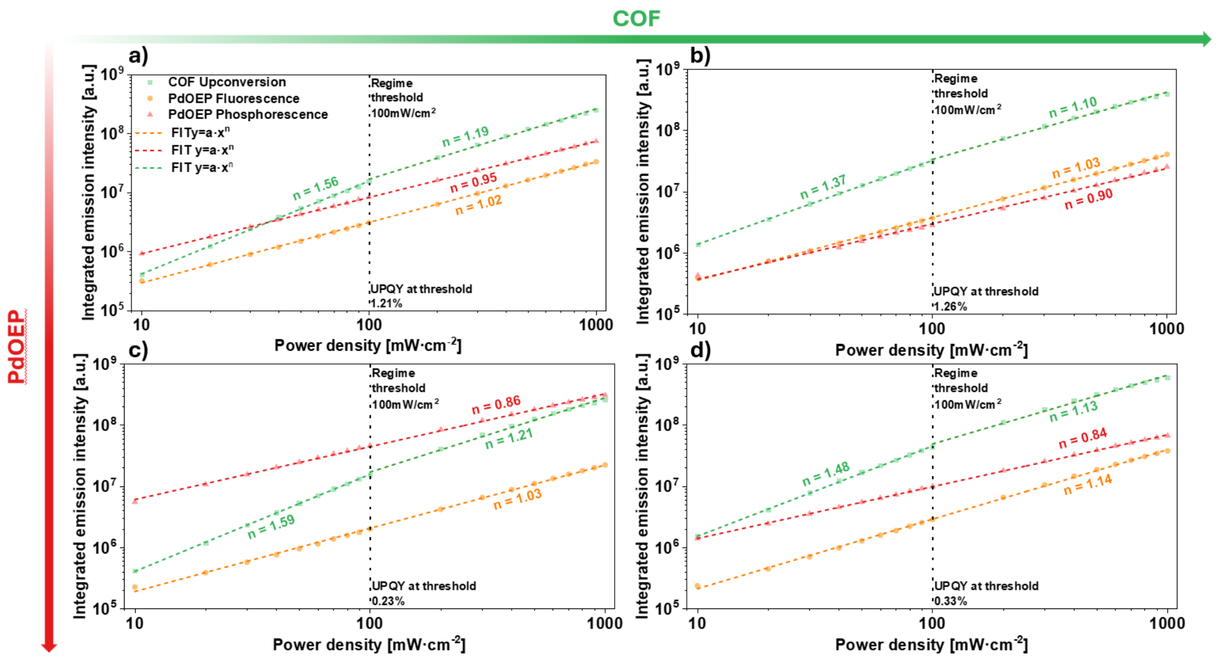

Figure S17. Double logarithmic plots representing integrated upconverted emission intensity of COF **Ant-COF-H** (green symbols), fluorescence (yellow symbols) and phosphorescence (red symbols) of **PdOEP** as a function of excitation power density at 523 nm and their linear fits. (a) 0.1x**Ant-COF-H** 0.1x**PdOEP**, (b) 1x**Ant-COF-H** 0.1x**PdOEP**, (c) 0.1x**Ant-COF-H** 1x**PdOEP**, (d) 1x**Ant-COF-H** 1x**PdOEP**.

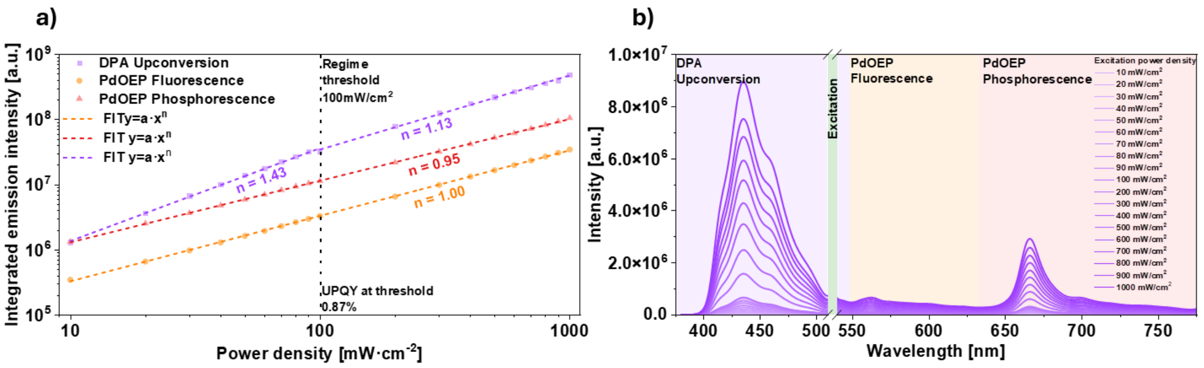

Figure S18. a) Double logarithmic plots representing integrated upconverted emission intensity of **DPA** (violet symbols), fluorescence (yellow symbols) and phosphorescence (red symbols) of **PdOEP** as a function of excitation power density at 523 nm and their linear fits, b) Photoluminescence spectra of **DPA** and **PdOEP** under excitation at 523 nm with power densities 10–1000 mW·cm^{-2}

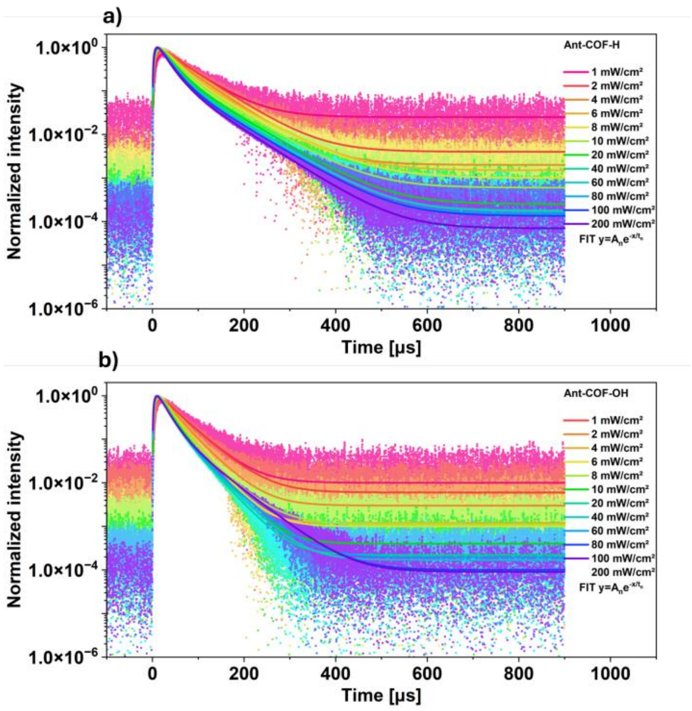

Figure S19. Time-dependent emission spectra of upconverting systems based on aminal-COFs and porphyrin sensitizer at different power densities of excitation laser beam at 523 nm: a) 0.1x**Ant-COF-H** 0.1x**PdOEP**, b) 0.1x**Ant-COF-OH** 0.1x**PdOEP**.

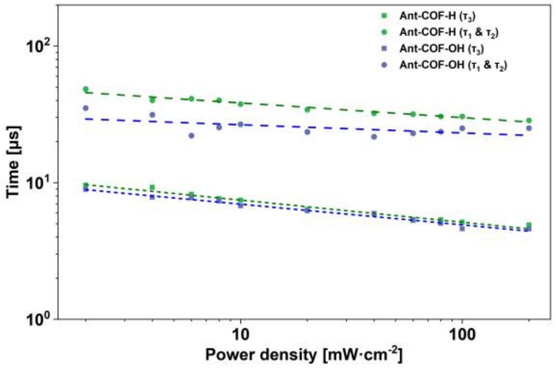

Figure S20. Extracted lifetimes (τ_1 – growth, τ_1 and τ_2 – decays) in systems based on **Ant-COF-H** and **Ant-COF-H** as a function of power density in double logarithmic scale. Decay times (τ_1 and τ_2) for both COFs were fitted monoexponentially with weighted amplitudes.

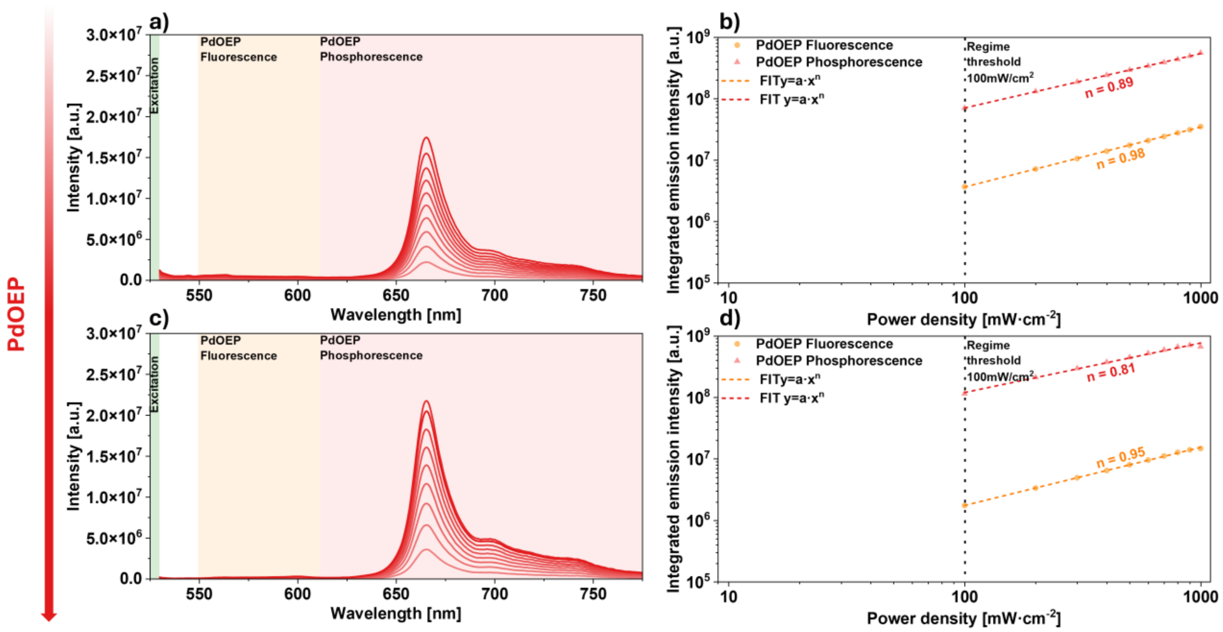

Figure S21. Fluorescence and phosphorescence spectra of **PdOEP** under two concentration regimes: a) 0.1x **PdOEP** and c) 1x **PdOEP** recorder at excitation power densities $100 - 100\, mW\ cm^{-2}$; double logarithmic plots representing **PdOEP** phosphorescence as a function of the excitation energy power density with linear fits.

Table S5. Measured quantum yields of upconversion $\boldsymbol{\Phi_{UC}}$, PdOEP fluorescence $\boldsymbol{\Phi_{F_S}}$ and PdOEP phosphorescence $\boldsymbol{\Phi_{P_S}}$, extracted quantum yields of triplet-triplet energy transfer $\boldsymbol{\Phi_{TTET}}$, and combined quantum yields of exciton energy transfer and triplet-triplet annihilation $\boldsymbol{\Phi_{EET} \cdot \Phi_{TTA}}$. The quantum yield of the intersystem crossing $\boldsymbol{\Phi_{ISC}}$ is assumed to be equal to 100%.

sample composition	Parameters					
	$\boldsymbol{\Phi_{UC}}$	$\boldsymbol{\Phi_{F_S}}$	$\boldsymbol{\Phi_{P_S}}$	$\boldsymbol{\Phi_{ISC}}$	$\boldsymbol{\Phi_{TTET}}$	$\boldsymbol{\Phi_{EET} \cdot \Phi_{TTA}}$
**Ant-COF-H** 0.1x **PdOEP** 0.1x	1.78 ± 0.25	0.26 ± 0.010	0.605 ± 0.016	100	44.521 ± 1.36	18.57 ± 2.99
**Ant-COF-H** 1x **PdOEP** 0.1x	1.66 ± 0.18	0.167 ± 0.011	0.110 ± 0.002	100	89.94 ± 0.49	8.58 ± 0.93
**Ant-COF-H** 0.1x **PdOEP** 1x	0.36 ± 0.06	0.033 ± 0.002	0.554 ± 0.067	100	7.31 ± 2.05	23.49 ± 3.29
**Ant-COF-H** 1.0x **PdOEP** 1x	0.47 ± 0.05	0.029 ± 0.003	0.062 ± 0.006	100	89.43 ± 0.47	2.45 ± 0.29
**DPA** **PdOEP** 0.1x	1.24 ± 0.18				72.02 ± 2.11	3.83 ± 0.66
**PdOEP** 0.1x	-	0.065 ± 0.002	1.091 ± 0.051	-	-	-
**PdOEP** 1x	-	0.011 ± 0.001	0.606 ± 0.058	-	-	-

11. Cartesian atomic coordinates

Table S6. Cartesian atom coordinates for the unit cell of **Ant-COF-H** with AB stacking.

P1									
a = 152 Å, b = 15.144 Å, c = 9.472 Å, α = 98.83°, β = 90.27°, γ = 94.12°									
	atom	x	y	z		atom	x	y	z
1	C	83.265	-56.861	-1.199	75	C	82.699	-50.039	3.481
2	N	84.509	-56.121	-1.133	76	N	83.943	-49.3	3.547
3	C	85.389	-56.324	-2.259	77	C	84.823	-49.503	2.42
4	C	85.219	-56.159	0.129	78	C	84.652	-49.338	4.809
5	C	86.419	-55.22	-2.265	79	C	85.853	-48.399	2.414
6	C	86.249	-55.055	0.124	80	C	85.682	-48.233	4.803
7	N	87.129	-55.258	-1.003	81	N	86.562	-48.436	3.677
8	H	82.934	-56.744	-2.241	82	H	82.367	-49.923	2.438
9	H	85.895	-57.304	-2.218	83	H	85.328	-50.482	2.461
10	H	84.8	-56.295	-3.173	84	H	84.233	-49.473	1.506

11	H	84.504	-56.032	0.941	85	H	83.938	-49.21	5.62
12	H	85.735	-57.119	0.284	86	H	85.169	-50.297	4.963
13	H	87.134	-55.348	-3.076	87	H	86.567	-48.526	1.603
14	H	85.902	-54.261	-2.419	88	H	85.336	-47.439	2.26
15	H	85.743	-54.076	0.083	89	H	85.177	-47.254	4.763
16	H	86.838	-55.085	1.037	90	H	86.272	-48.263	5.717
17	C	83.888	-48.266	-0.841	91	C	83.322	-41.444	3.839
18	C	84.711	-49.427	-1.027	92	C	84.144	-42.606	3.652
19	C	82.498	-48.444	-0.586	93	C	81.931	-41.622	4.093
20	C	84.09	-50.708	-0.958	94	C	83.524	-43.886	3.721
21	C	81.945	-49.687	-0.525	95	C	81.378	-42.866	4.154
22	C	82.753	-50.832	-0.729	96	C	82.187	-44.01	3.95
23	C	84.46	-46.99	-0.89	97	C	83.893	-40.169	3.789
24	C	85.839	-46.847	-1.107	98	C	85.273	-40.025	3.572
25	C	86.09	-49.284	-1.244	99	C	85.524	-42.462	3.435
26	C	86.662	-48.008	-1.294	100	C	86.096	-41.186	3.386
27	C	86.459	-45.566	-1.176	101	C	85.893	-38.744	3.503
28	C	87.797	-45.442	-1.405	102	C	87.23	-38.621	3.274
29	C	88.052	-47.83	-1.547	103	C	87.486	-41.009	3.132
30	C	88.605	-46.587	-1.609	104	C	88.039	-39.765	3.07
31	C	83.618	-45.769	-0.856	105	C	83.051	-38.948	3.823
32	C	83.351	-45.047	0.305	106	C	82.785	-38.225	4.984
33	C	83.171	-45.262	-2.073	107	C	82.604	-38.441	2.607
34	C	82.752	-43.795	0.238	108	C	82.185	-36.973	4.917
35	C	82.583	-44.013	-2.139	109	C	82.016	-37.191	2.541
36	C	82.423	-43.236	-0.996	110	C	81.857	-36.414	3.683
37	H	81.888	-47.566	-0.426	111	H	81.321	-40.745	4.253
38	H	84.705	-51.589	-1.086	112	H	84.139	-44.767	3.593
39	H	80.89	-49.81	-0.319	113	H	80.323	-42.988	4.36
40	H	82.297	-51.812	-0.694	114	H	81.731	-44.991	3.985
41	H	85.845	-44.685	-1.049	115	H	85.278	-37.864	3.631
42	H	88.253	-44.462	-1.441	116	H	87.686	-37.64	3.239
43	H	88.663	-48.708	-1.707	117	H	88.096	-41.886	2.972
44	H	89.661	-46.464	-1.814	118	H	89.094	-39.643	2.865
45	H	83.664	-45.443	1.264	119	H	83.097	-38.621	5.943
46	H	83.339	-45.83	-2.977	120	H	82.772	-39.008	1.702
47	H	82.583	-43.229	1.143	121	H	82.016	-36.407	5.823
48	H	82.31	-43.602	-3.101	122	H	81.743	-36.78	1.578
49	C	86.932	-50.505	-1.278	123	C	86.366	-43.684	3.401
50	C	87.198	-51.227	-2.44	124	C	86.632	-44.405	2.239
51	C	87.378	-51.013	-0.062	125	C	86.812	-44.191	4.617

52	C	87.798	-52.48	-2.373	126	C	87.231	-45.658	2.307
53	C	87.966	-52.262	0.004	127	C	87.4	-45.441	4.683
54	C	88.126	-53.039	-1.139	128	C	87.56	-46.217	3.54
55	C	88.373	-54.518	-0.937	129	C	87.806	-47.696	3.743
56	N	89.309	-55.113	-1.876	130	N	88.743	-48.291	2.803
57	H	86.886	-50.831	-3.398	131	H	86.32	-44.009	1.281
58	H	87.21	-50.446	0.843	132	H	86.644	-43.624	5.522
59	H	87.966	-53.045	-3.279	133	H	87.4	-46.224	1.401
60	H	88.239	-52.674	0.966	134	H	87.672	-45.852	5.645
61	H	88.704	-54.635	0.106	135	H	88.137	-47.813	4.785
62	N	91.48	-56.266	-0.259	136	N	90.914	-49.445	4.42
63	C	91.341	-54.819	-0.479	137	C	90.775	-47.997	4.2
64	C	90.164	-56.898	-0.353	138	C	89.597	-50.076	4.326
65	C	90.626	-54.481	-1.782	139	C	90.059	-47.659	2.897
66	C	89.448	-56.56	-1.656	140	C	88.882	-49.739	3.023
67	H	90.796	-54.413	0.377	141	H	90.23	-47.592	5.056
68	H	92.334	-54.372	-0.483	142	H	91.767	-47.55	4.196
69	H	90.285	-57.977	-0.247	143	H	89.719	-51.155	4.432
70	H	89.567	-56.569	0.501	144	H	89.001	-49.748	5.18
71	H	90.504	-53.402	-1.888	145	H	89.938	-46.581	2.792
72	H	91.222	-54.809	-2.636	146	H	90.656	-47.988	2.043
73	H	89.994	-56.966	-2.512	147	H	89.427	-50.144	2.167
74	H	88.456	-57.007	-1.652	148	H	87.889	-50.185	3.028

Table S7. Cartesian atom coordinates for the unit cell of **Ant-COF-OH** with AB stacking.

P1									
a = 8.980 Å, b = 15.788 Å, c = 9.929 Å, α = 98.07°, β = 93.21°, γ = 91.45°									
	atom	x	y	z		atom	x	y	z
1	C	75.967	-61.451	-1.276	77	C	75.524	-54.188	3.618
2	N	77.15	-60.628	-1.129	78	N	76.708	-53.367	3.761
3	C	77.992	-60.642	-2.306	79	C	77.553	-53.388	2.587
4	C	77.925	-60.836	0.082	80	C	77.48	-53.572	4.975
5	C	79.024	-59.548	-2.209	81	C	78.585	-52.293	2.683
6	C	78.958	-59.742	0.179	82	C	78.512	-52.479	5.071
7	N	79.8	-59.755	-0.998	83	N	79.357	-52.497	3.897
8	H	75.634	-61.275	-2.305	84	H	75.191	-54.017	2.588
9	H	78.499	-61.612	-2.424	85	H	78.062	-54.358	2.477
10	H	77.369	-60.48	-3.184	86	H	76.933	-53.233	1.706
11	H	77.272	-60.802	0.954	87	H	76.824	-53.534	5.846
12	H	78.429	-61.814	0.082	88	H	77.982	-54.55	4.981
13	H	79.677	-59.583	-3.081	89	H	79.241	-52.332	1.812
14	H	78.521	-58.57	-2.21	90	H	78.082	-51.315	2.677
15	H	78.449	-58.772	0.296	91	H	78.005	-51.508	5.184
16	H	79.581	-59.901	1.057	92	H	79.133	-52.636	5.951
17	C	76.917	-52.193	-0.732	93	C	76.456	-44.934	4.206
18	C	77.533	-53.48	-0.889	94	C	77.068	-46.223	4.04
19	C	75.542	-52.139	-0.374	95	C	75.086	-44.879	4.58
20	C	76.721	-54.64	-0.738	96	C	76.252	-47.381	4.182
21	C	74.809	-53.277	-0.207	97	C	74.352	-46.016	4.748
22	C	75.401	-54.545	-0.414	98	C	74.935	-47.284	4.517
23	C	77.668	-51.027	-0.94	99	C	77.207	-43.769	3.986
24	C	79.029	-51.118	-1.267	100	C	78.566	-43.863	3.656
25	C	78.893	-53.572	-1.218	101	C	78.429	-46.317	3.713
26	C	79.642	-52.406	-1.435	102	C	79.181	-45.151	3.503
27	C	79.844	-49.959	-1.409	103	C	79.379	-42.704	3.499
28	C	81.161	-50.055	-1.746	104	C	80.698	-42.801	3.173
29	C	81.012	-52.46	-1.809	105	C	80.555	-45.207	3.145
30	C	81.745	-51.323	-1.977	106	C	81.289	-44.071	2.971
31	C	77.006	-49.701	-0.961	107	C	76.546	-42.442	3.959
32	C	76.522	-49.067	0.176	108	C	76.069	-41.797	5.093
33	C	76.862	-49.079	-2.201	109	C	76.402	-41.828	2.715
34	C	75.938	-47.802	0.089	110	C	75.488	-40.531	4.998
35	C	76.307	-47.817	-2.281	111	C	75.855	-40.563	2.628
36	C	75.872	-47.142	-1.147	112	C	75.425	-39.879	3.757
37	H	75.076	-51.173	-0.243	113	H	74.625	-43.913	4.726

38	H	77.171	-55.607	-0.91	114	H	76.697	-48.348	3.993
39	H	73.763	-53.203	0.052	115	H	73.31	-45.94	5.025
40	H	74.791	-55.436	-0.336	116	H	74.321	-48.173	4.587
41	H	79.399	-48.993	-1.22	117	H	78.929	-41.736	3.667
42	H	81.775	-49.166	-1.816	118	H	81.308	-41.911	3.089
43	H	81.474	-53.426	-1.955	119	H	81.022	-46.174	3.017
44	H	82.787	-51.397	-2.255	120	H	82.335	-44.146	2.711
45	H	76.61	-49.529	1.151	121	H	76.16	-42.251	6.071
46	H	77.219	-49.577	-3.091	122	H	76.752	-42.334	1.826
47	H	76.239	-47.319	-3.241	123	H	75.789	-40.071	1.665
48	C	79.555	-54.898	-1.192	124	C	79.091	-47.643	3.734
49	C	80.03	-55.543	-2.328	125	C	79.571	-48.277	2.595
50	C	79.706	-55.51	0.052	126	C	79.241	-48.264	4.973
51	C	80.612	-56.808	-2.233	127	C	80.161	-49.539	2.681
52	C	80.255	-56.774	0.138	128	C	79.8	-49.525	5.051
53	C	80.681	-57.46	-0.993	129	C	80.232	-50.199	3.916
54	C	80.983	-58.933	-0.855	130	C	80.539	-51.673	4.043
55	N	82.007	-59.376	-1.82	131	N	81.561	-52.108	3.074
56	H	79.936	-55.089	-3.306	132	H	79.477	-47.816	1.62
57	H	79.359	-55.003	0.941	133	H	78.888	-47.766	5.864
58	H	80.325	-57.265	1.101	134	H	79.871	-50.022	6.011
59	H	81.316	-59.105	0.175	135	H	80.873	-51.851	5.072
60	N	83.924	-61.015	-0.308	136	N	83.481	-53.745	4.583
61	C	83.856	-59.552	-0.168	137	C	83.417	-52.281	4.721
62	C	82.603	-61.527	-0.711	138	C	82.16	-54.258	4.182
63	C	83.329	-58.865	-1.417	139	C	82.883	-51.595	3.474
64	C	82.071	-60.839	-1.958	140	C	81.629	-53.571	2.933
65	H	83.217	-59.344	0.69	141	H	82.786	-52.068	5.584
66	H	84.845	-59.169	0.065	142	H	84.408	-51.9	4.943
67	H	82.683	-62.603	-0.879	143	H	82.241	-55.334	4.016
68	H	81.912	-61.382	0.123	144	H	81.468	-54.112	5.014
69	H	83.252	-57.789	-1.254	145	H	82.804	-50.519	3.638
70	H	84.021	-59.014	-2.25	146	H	83.572	-51.743	2.639
71	H	82.702	-61.052	-2.821	147	H	82.268	-53.778	2.074
72	H	81.079	-61.22	-2.181	148	H	80.641	-53.956	2.703
73	O	81.084	-57.388	-3.36	149	O	75.013	-39.952	6.123
74	O	75.461	-47.233	1.219	150	O	80.636	-50.106	1.549
75	H	75.049	-46.366	0.944	151	H	74.603	-39.087	5.84
76	H	81.498	-58.251	-3.077	152	H	81.05	-50.972	1.822

12. Kinetic model parameters

Table S8. Input parameters used for the CW steady-state TTET–TTA upconversion model (PdOEP/DPA in toluene).

Parameter	Symbol	Code variable	Value	Comment
Sensitizer concentration	c_S	c_S_mM	$0.017\ m\mathrm{M}$[a]	Used in Eq. (S6) and bounds $[T_S] \leq c_S$.
Annihilator concentration	c_A	c_A_mM	$0.032\ m\mathrm{M}$[a]	Used in Eq. (S17) and bounds $[T_A] \leq c_A$.
Excitation wavelength	λ	lambda_exc_nm	$535.0\ nm$[a]	Used in Eq. (S7)
Optical path length	ℓ	path_length_cm	$0.10\ cm$[a]	Used in Eq. (S6) and Eq. (S7)
Sensitizer molar absorption coefficient	ε	epsilon_S_M_inv_cm_inv	$4.5 \cdot 10^4 \mathrm{M}^{-1} cm^{-1}$[b]	Used in Eq. (S6)
Absorbed fraction (computed)	f_{abs}	f_abs	(computed)	From Eq. (S6)
Absorption rate	$R_{\mathrm{abs}}(P)$	Rabs	(computed)	From Eq. (S7)
ISC yield	ϕ_{ISC}	phi_ISC	1.0[c]	Used in Eq. (S8)
Sensitizer triplet lifetime	τ_{T_S}	tau_T_S_us	$270\ \mu s$[d]	Used in Eq. (S12)
Annihilator triplet lifetime	τ_{T_A}	tau_T_A_us	$8610\ \mu s$[e]	Used in Eq. (S12)
Extra sensitizer triplet quenching	$k_{q,S}$	kq_S_s_inv	$0\ s^{-1}$	Used in Eq . (S12)
Extra annihilator triplet quenching	$k_{q,A}$	kq_A_s_inv	$0\ s^{-1}$	Used in Eq. (S12)
Effective sensitizer loss	k_{T_S}	k_T_S_eff	(computed)	From Eq. (S12)

Effective annihilator loss	k_{T_A}	k_T_A_eff	(computed)	From Eq. (S12)
Temperature	T	T_K	$298.15\ K$	Used in Eq. (S9).
Viscosity	η	viscosity_mPa_s	$0.59\ mPa \cdot s$[f]	Used in Eq. (S9).
TTET hydrodynamic radius (sensitizer)	$r_{S,\mathrm{TTET}}$	r_S_TTET_nm	$0.1\ nm$	Used in Eq. (S9)
TTET hydrodynamic radius (annihilator)	$r_{A,\mathrm{TTET}}$	r_A_TTET_nm	$0.5\ nm$	Used in Eq. (S9)
TTA hydrodynamic radius (partner A1)	$r_{A1,\mathrm{TTA}}$	r_A1_TTA_nm	$0.5\ nm$	Used in Eq. (S9)
TTA hydrodynamic radius (partner A2)	$r_{A2,\mathrm{TTA}}$	r_A2_TTA_nm	$0.5\ nm$	Used in Eq. (S9)
TTET capture radius (contact default)	R_{TTET}	R_TTET_nm_eff	(computed)<br>$r_{S,\mathrm{TTET}} + r_{A,\mathrm{TTET}} =$ 0.6nm	Used in Eq. (S10)
TTA capture radius (contact default)	R_{TTA}	R_TTA_nm_eff	(computed)<br>$r_{A1,\mathrm{TTA}} + r_{A2,\mathrm{TTA}} =$ 1.0nm	Used in Eq. (S10)
Intrinsic TTET rate	$k_{\mathrm{intr,TTET}}$	k_TTET_intr_M_inv_s	$1.53 \cdot 10^9 \mathrm{M}^{-1} s^{-1}$[g]	Used in Eq. (S11)
Intrinsic TTA rate	$k_{\mathrm{intr,TTA}}$	k_TTA_intr_M_inv_s	$7.2 \cdot 10^8 \mathrm{M}^{-1} s^{-1}$[h]	Used in Eq. (S11)
Diffusion-limited TTET rate (computed)	$k_{\mathrm{diff,TTET}}$	k_diff_TTET	(computed)	From Eq. (S10)
Diffusion-limited TTA rate (computed)	$k_{\mathrm{diff,TTA}}$	k_diff_TTA	(computed)	From Eq. (S10)

Effective TTET rate (computed)	k_{TTET}	k_TTET_eff.	(computed)	From Eq. (S11)
Effective TTA rate (computed)	k_{TTA}	k_TTA_eff	(computed)	From Eq. (S11)
Fluorescence quantum yield	ϕ_F	phi_F	0.86[i]	Used in Eq. (S20).
Internal factor in TTA→S_1	η_{ET}	eta_ET	1.0	Used in Eq. (S19).
TTA channel efficiency	η_{TTA}	eta_TTA	0.03	Used in Eq. (S19).
Spin-statistical singlet fraction	f_{singlet}	f_singlet	$1/9 = 0.1111$	Used in Eq. (S19).
Composite singlet yield	$\eta_{\mathrm{TTA}\rightarrow S_1}$	eta_TTA_to_S1	(computed)	From Eq. (S19)
Power density grid	P	P_min_mW_cm2 → P_max_mW_cm2	$0.1 \rightarrow 10000$ $mWcm^{-2}$	Log-spaced grid for $I_{\mathrm{UC}}(P)$ and $n(P)$.
Number of power points	N_P	P_points	100	Resolution of $n(P)$
Fixed-point iterations	N_{iter}	n_iter_freeA	10	Convergence of coupled Eqs. (S14)–(S17).

[a] Own data
[b] From Ref.[73]
[c] From Ref.[74]
[d] From Ref.[75]
[e] From Ref.[76]
[f] From Ref.[77]
[g] From Ref.[78]
[h] From Ref.[79]
[i] From Ref.[80]

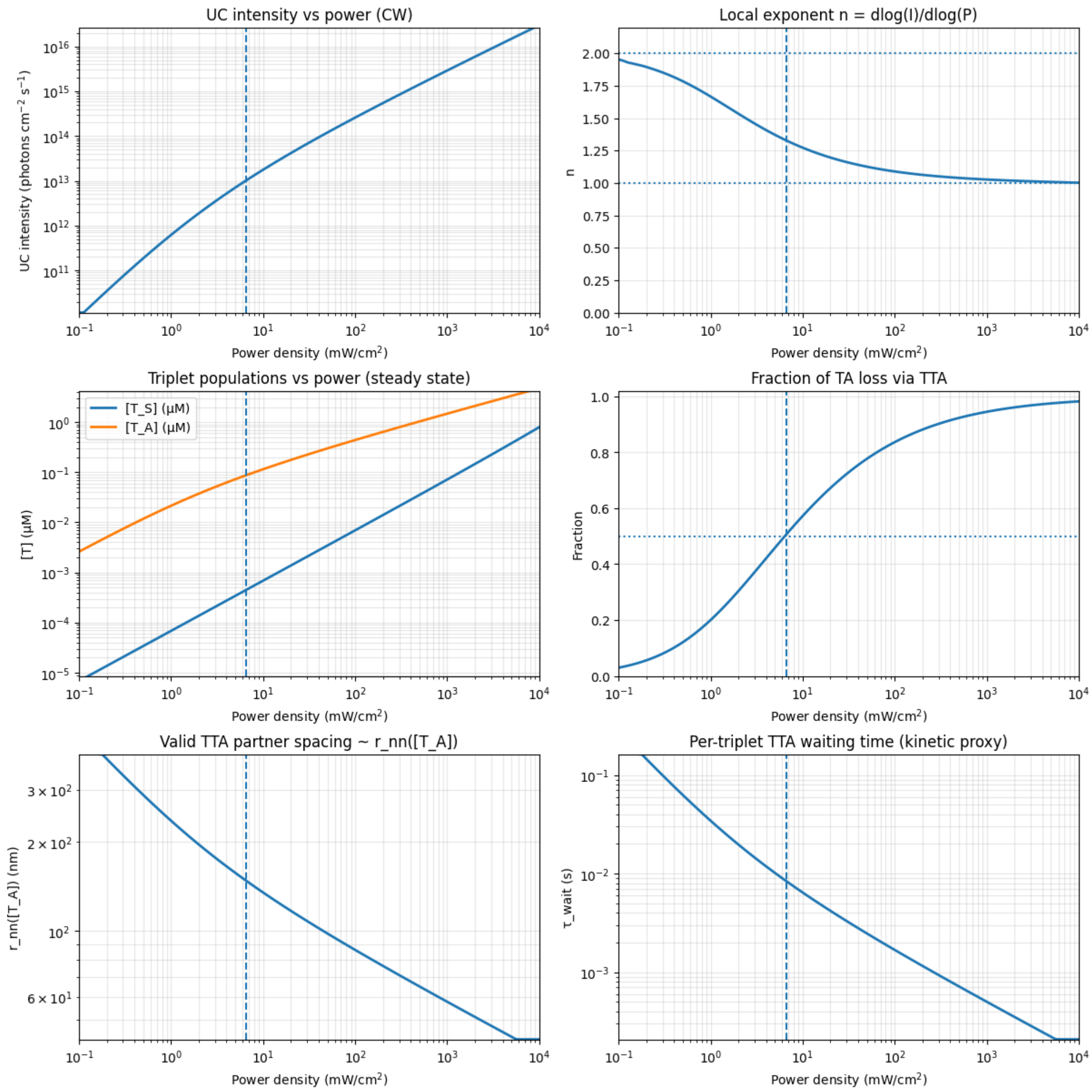

Figure 22. Continuous-wave (CW) power-dependence predicted by the mixed-order TTET–TTA steady-state model for the PdOEP/DPA system (Beer–Lambert absorption and diffusion-influenced bimolecular rate constants). **Top left:** simulated upconversion intensity $I_{\mathrm{UC}}(P)$ (photon flux, photons $cm^{-2}\ s^{-1}$) as a function of excitation power density $P(mW\ cm^{-2})$ on log–log axes. **Top right:** local apparent power-law exponent $n(P) = \mathrm{dlog}\ I_{\mathrm{UC}}/\mathrm{dlog}\ P$, illustrating the crossover from a near-quadratic regime at low power ($n \approx 2$, first-order triplet loss dominated) to an approximately linear regime at high power ($n \to 1$, TTA-dominated). **Middle left:** steady-state sensitizer and annihilator triplet concentrations $[T_S]$and $[T_A]$ as a function of P. **Middle right:** fraction of annihilator-triplet loss occurring via TTA, $\frac{2k_{\mathrm{TTA}}[T_A]^2}{k_{T_A}[T_A]+2k_{\mathrm{TTA}}[T_A]^2}$, which approaches unity as the bimolecular pathway becomes dominant. **Bottom left:** estimated mean spacing between excited annihilator triplets (valid TTA partners), $r_{\mathrm{nn}}([T_A])$. **Bottom right:** corresponding per-triplet TTA waiting time $\tau_{\mathrm{wait}} \approx [2k_{\mathrm{TTA}}[T_A]]^{-1}$. The vertical dashed line indicates the model-defined

threshold (power at which first- and second-order annihilator-triplet loss contributions are equal; fraction via $TTA \approx 0.5$), while horizontal dotted lines mark the reference slopes $n = 2$ and $n = 1$.

13. Literature

[55] J. Rodríguez-Carvajal, "Recent advances in magnetic structure determination by neutron powder diffraction" *Physica B* **1993**, *192*, 55–69.
[56] M. Thommes, K. Kaneko, A. V. Neimark, J. P. Olivier, F. Rodriguez-Reinoso, J. Rouquerol, K. S. W. Sing, "Physisorption of gases, with special reference to the evaluation of surface area and pore size distribution (IUPAC Technical Report)" *Pure Appl. Chem.* **2015**, *87*, 1051–1069.
[57] J. W. M. Osterrieth, J. Rampersad, D. Madden, N. Rampal, L. Skoric, B. Connolly, M. D. Allendorf, V. Stavila, J. L. Snider, R. Ameloot, J. Marreiros, C. Ania, D. Azevedo, E. Vilarrasa-Garcia, B. F. Santos, X. Bu, Z. Chang, H. Bunzen, N. R. Champness, S. L. Griffin, B. Chen, R. Lin, B. Coasne, S. Cohen, J. C. Moreton, Y. J. Colón, L. Chen, R. Clowes, F. Coudert, Y. Cui, B. Hou, D. M. D'Alessandro, P. W. Doheny, M. Dincă, C. Sun, C. Doonan, M. T. Huxley, J. D. Evans, P. Falcaro, R. Ricco, O. Farha, K. B. Idrees, T. Islamoglu, P. Feng, H. Yang, R. S. Forgan, D. Bara, S. Furukawa, E. Sanchez, J. Gascon, S. Telalović, S. K. Ghosh, S. Mukherjee, M. R. Hill, M. M. Sadiq, P. Horcajada, P. Salcedo-Abraira, K. Kaneko, R. Kukobat, J. Kenvin, S. Keskin, S. Kitagawa, K. Otake, R. P. Lively, S. J. A. DeWitt, P. Llewellyn, B. V. Lotsch, S. T. Emmerling, A. M. Pütz, C. Martí-Gastaldo, N. M. Padial, J. García-Martínez, N. Linares, D. Maspoch, J. A. Suárez Del Pino, P. Moghadam, R. Oktavian, R. E. Morris, P. S. Wheatley, J. Navarro, C. Petit, D. Danaci, M. J. Rosseinsky, A. P. Katsoulidis, M. Schröder, X. Han, S. Yang, C. Serre, G. Mouchaham, D. S. Sholl, R. Thyagarajan, D. Siderius, R. Q. Snurr, R. B. Goncalves, S. Telfer, S. J. Lee, V. P. Ting, J. L. Rowlandson, T. Uemura, T. Iiyuka, M. A. Van Der Veen, D. Rega, V. Van Speybroeck, S. M. J. Rogge, A. Lamaire, K. S. Walton, L. W. Bingel, S. Wuttke, J. Andreo, O. Yaghi, B. Zhang, C. T. Yavuz, T. S. Nguyen, F. Zamora, C. Montoro, H. Zhou, A. Kirchon, D. Fairen-Jimenez, "How Reproducible are Surface Areas Calculated from the BET Equation?" *Adv. Mater.* **2022**, *34*, 2201502.
[58] J. VandeVondele, M. Krack, F. Mohamed, M. Parrinello, T. Chassaing, J. Hutter, "Quickstep: Fast and accurate density functional calculations using a mixed Gaussian and plane waves approach" *Comput. Phys. Commun.* **2005**, *167*, 103–128.
[59] T. D. Kühne, M. Iannuzzi, M. Del Ben, V. V. Rybkin, P. Seewald, F. Stein, T. Laino, R. Z. Khaliullin, O. Schütt, F. Schiffmann, D. Golze, J. Wilhelm, S. Chulkov, M. H. Bani-Hashemian, V. Weber, U. Borštnik, M. Taillefumier, A. S. Jakobovits, A. Lazzaro, H. Pabst, T. Müller, R. Schade, M. Guidon, S. Andermatt, N. Holmberg, G. K. Schenter, A. Hehn, A. Bussy, F. Belleflamme, G. Tabacchi, A. Glöß, M. Lass, I. Bethune, C. J. Mundy, C. Plessl, M. Watkins, J. VandeVondele, M. Krack, J. Hutter, "CP2K: An electronic structure and molecular dynamics software package - Quickstep: Efficient and accurate electronic structure calculations" *J. Chem. Phys.* **2020**, *152*, 194103.
[60] J. P. Perdew, K. Burke, M. Ernzerhof, "Generalized Gradient Approximation Made Simple" *Phys. Rev. Lett.* **1996**, *77*, 3865–3868.
[61] S. Grimme, S. Ehrlich, L. Goerigk, "Effect of the damping function in dispersion corrected density functional theory" *J. Comput. Chem.* **2011**, *32*, 1456–1465.
[62] S. Goedecker, M. Teter, J. Hutter, "Separable dual-space Gaussian pseudopotentials" *Phys. Rev. B* **1996**, *54*, 1703–1710.
[63] J. VandeVondele, J. Hutter, "Gaussian basis sets for accurate calculations on molecular systems in gas and condensed phases" *J. Chem. Phys.* **2007**, *127*, 114105.
[64] C. Adamo, V. Barone, "Toward reliable density functional methods without adjustable parameters: The PBE0 model" *J. Chem. Phys.* **1999**, *110*, 6158–6170.
[65] T. F. Willems, C. H. Rycroft, M. Kazi, J. C. Meza, M. Haranczyk, "Algorithms and tools for high-throughput geometry-based analysis of crystalline porous materials" *Microporous Mesoporous Mater.* **2012**, *149*, 134–141.

[66] M. Gutsche, J. Podlech, “Synthesis of Octahydroperylene, the Framework of Altertoxin III” *Eur. J. Org. Chem.* **2024**, *27*, e202301053.
[67] N. G. Barnes, A. W. Parker, A. A. Ahmed Mal Ullah, P. A. Ragazzon, J. A. Hadfield, “A 2-step synthesis of Combretastatin A-4 and derivatives as potent tubulin assembly inhibitors” *Bioorg. Med. Chem.* **2020**, *28*, 115684.
[68] J. Grajewski, M. Zgorzelak, A. Janiak, K. Taras-Goślińska, “Controlled, Sunlight-Driven Reversible Cycloaddition of Multiple Singlet Oxygen Molecules to Anthracene-Containing Trianglimine Macrocycles” *ChemPlusChem* **2022**, *87*, e202100510.
[69] M. T. Chaudhry, S. Ota, F. Lelj, M. J. MacLachlan, “Breathing Room: Restoring Free Rotation in a Schiff-Base Macrocycle through Endoperoxide Formation” *Org. Lett.* **2021**, *23*, 9538–9542.
[70] J. Rodríguez-Carvajal, “Recent advances in magnetic structure determination by neutron powder diffraction” *Physica B* **1993**, *192*, 55–69.
[71] M. Thommes, K. Kaneko, A. V. Neimark, J. P. Olivier, F. Rodriguez-Reinoso, J. Rouquerol, K. S. W. Sing, “Physisorption of gases, with special reference to the evaluation of surface area and pore size distribution (IUPAC Technical Report)” *Pure Appl. Chem.* **2015**, *87*, 1051–1069.
[72] S. Grimme, S. Ehrlich, L. Goerigk, “Effect of the damping function in dispersion corrected density functional theory” *J. Comput. Chem.* **2011**, *32*, 1456–1465.
[73] C. H. Mulyadi, M. Uji, B. Parmar, K. Orihashi, N. Yanai, “Triplet–Triplet Annihilation-Based Photon Upconversion with a Macrocyclic Parallel Dimer” *Precis. Chem.* **2024**, *2*, 539–544.
[74] M. Adams, N. Baroni, M. Oldenburg, F. Kraffert, J. Behrends, R. W. MacQueen, R. Haldar, D. Busko, A. Turshatov, G. Emandi, M. O. Senge, C. Wöll, K. Lips, B. S. Richards, I. A. Howard, “Reaction of porphyrin-based surface-anchored metal–organic frameworks caused by prolonged illumination” *Phys. Chem. Chem. Phys.* **2018**, *20*, 29142–29151.
[75] M. Banaszek, B. Golec, R. Rybakiewicz-Sekita, J. Kowalski, P. Szczodry, N. Dutkiewicz, J. Waluk, A. Gorski, “Enhancing Photostability by Thermodynamic and Kinetic Factors: Free-Base and Palladium *meso* -Aryl-octaethylporphyrins” *J. Phys. Chem. B* **2025**, *129*, 5491–5500.
[76] V. Gray, D. Dzebo, A. Lundin, J. Alborzpour, M. Abrahamsson, B. Albinsson, K. Moth-Poulsen, “Photophysical characterization of the 9,10-disubstituted anthracene chromophore and its applications in triplet–triplet annihilation photon upconversion” *J. Mater. Chem. C* **2015**, *3*, 11111–11121.
[77] viscosity of toluene can be found under https://wiki.anton-paar.com/en/toluene, **2026** (accessed: 19.01.2026).
[78] M. Lindgren, V. M. Bjelland, T.-B. Melø, C. McCracken, S. Seo, H. Nakashima, “The Triplet–Triplet Annihilation Efficiency of Some 9,10-Substituted Diphenyl Anthracene Variants—A Decisive Analysis from Kinetic Rate Constants” *Optics* **2025**, *6*, 8.
[79] F. Edhborg, A. Olesund, B. Albinsson, “Best practice in determining key photophysical parameters in triplet–triplet annihilation photon upconversion” *Photochem. Photobiol. Sci.* **2022**, *21*, 1143–1158.
[80] A. Demeter, “First Steps in Photophysics. I. Fluorescence Yield and Radiative Rate Coefficient of 9,10-Bis(phenylethynyl)anthracene in Paraffins” *J. Phys. Chem. A* **2014**, *118*, 9985–9993.
